# Microglial TREM-1 receptor mediates neuroinflammatory injury via interaction with SYK in experimental ischemic stroke

**DOI:** 10.1038/s41419-019-1777-9

**Published:** 2019-07-19

**Authors:** Pengfei Xu, Xiaohao Zhang, Qian Liu, Yi Xie, Xiaolei Shi, Jingjing Chen, Yunzi Li, Hongquan Guo, Rui Sun, Ye Hong, Xinfeng Liu, Gelin Xu

**Affiliations:** 1 Department of Neurology, Jinling Hospital, Medical School of Nanjing University, Nanjing, 210002 Jiangsu China; 2 Department of Neurology, The First Affiliated Hospital of USTC, Division of Life Sciences and Medicine, University of Science and Technology of China, Hefei, 230001 Anhui China; 3 Department of Neurology, Jiangsu Provincial Second Chinese Medicine Hospital, The Second Affiliated Hospital of Nanjing University of Chinese Medicine, Nanjing, 210002 Jiangsu China; 4 Department of Neurology, Yijishan Hospital, The First Affiliated Hospital of Wannan Medical College, Wuhu, 241001 Anhui China; 5 Department of Neurology, Jinling Clinical College of Nanjing Medical University, Nanjing, 210002 Jiangsu China; 6 Department of Neurology, Jinling Hospital, Southern Medical University, Nanjing, 210002 Jiangsu China

**Keywords:** Cell death, Cell death, Cell death in the nervous system, Cell death in the nervous system

## Abstract

Neuroinflammation is initiated in response to ischemic stroke, generally with the hallmarks of microglial activation and collateral brain injury contributed by robust inflammatory effects. Triggering receptor expressed on myeloid cells (TREM)-1, an amplifier of the innate immune response, is a critical regulator of inflammation. This study identified that microglial TREM-1 expression was upregulated following cerebral ischemic injury. After pharmacologic inhibition of TREM-1 with synthetic peptide LP17, ischemia-induced infarction and neuronal injury were substantially alleviated. Moreover, blockade of TREM-1 can potentiate cellular proliferation and synaptic plasticity in hippocampus, resulting in long-term functional improvement. Microglial M1 polarization and neutrophil recruitment were remarkably abrogated as mRNA levels of M1 markers, chemokines, and protein levels of myeloperoxidase and intracellular adhesion molecule-1 (ICAM-1) were decreased by LP17. Mechanistically, both in vivo and in vitro, we delineated that TREM-1 can activate downstream pro-inflammatory pathways, CARD9/NF-κB, and NLRP3/caspase-1, through interacting with spleen tyrosine kinase (SYK). In addition, TREM-1-induced SYK initiation was responsible for microglial pyroptosis by elevating levels of gasdermin D (GSDMD), N-terminal fragment of GSDMD (GSDMD-N), and forming GSDMD pores, which can facilitate the release of intracellular inflammatory factors, in microglia. In summary, microglial TREM-1 receptor yielded post-stroke neuroinflammatory damage via associating with SYK.

## Introduction

In the central nervous system (CNS), microglia serve as the resident mononuclear phagocytes and are key cellular mediators participated in both acute and chronic neuroinflammatory responses^1^. Neuroinflammation plays a dual role in cerebral ischemic cascade^2^. It is an essential tool to eliminate dead cells and necrotic debris caused by cerebral flow reduction and subsequent reperfusion insult. However, excessive inflammatory responses can be detrimental, and may lead to increased infarction and reduced neuronal plasticity^3,4^. Interestingly, in clinical trials, anti-inflammatory therapy has not shown the expected results in cerebral ischemia^5,6^. Therefore, it is necessary to gain a deeper understanding of neuroinflammation after ischemic stroke.

Triggering receptor expressed on myeloid cells 1 (TREM-1), a transmembrane immune receptor, is expressed on the surface of phagocytic cells, acting as a potent amplifier of inflammation^7–10^. TREM-1 can recruit spleen tyrosine kinase (SYK)^9^, which has been regarded as a launching point of inflammation after ischemic stroke^11^. Clinical studies have detected sTREM-1, a soluble form of TREM-1, in the serum of patients with inflammatory conditions^12–15^. Blockade of TREM-1 activation by fusion proteins or synthetic inhibitory peptides can inhibit pro-inflammatory mediators’ production and limit leukocytes recruitment^15–18^. However, to date, the precise role of TREM-1 and its downstream factors orchestrating post-stroke neuroinflammation has not been clarified. Herein, in this study, using mice middle cerebral artery occlusion (MCAO) model and microglia oxygen-glucose deprivation (OGD) model, we examined whether microglial TREM-1 regulates neuroinflammation following brain ischemic injury and whether this involvement is though interaction with SYK.

## Methods and materials

### Animals and ethical statement

Adult male C57BL/6J mice (20–25 g) were purchased from Model Animal Research Center of Nanjing University and housed in individual cages with a 12 h light/dark cycle and given free access to food and water. All experiments were approved by Jinling Hospital Animal Care Committee and were implemented according to the National Institute of Health Guide for the Care and Use of Laboratory Animals (NIH Publications no. 80-23, revised 1996). At least five mice were analyzed for each data point.

### Surgical procedures

Mice cerebral ischemia/reperfusion (I/R) models were induced by MCAO surgery using the intraluminal filament following previous method^19,20^. In brief, mice were anesthetized with 2% isoflurane in O_2_ (RWD Life Science, China). The right external carotid artery (ECA) was ligated and a silicon-coated monofilament (diameter: 0.18 ± 0.01 mm, Beijing Cinontech Co., Ltd, China) was inserted into ECA and advanced into internal carotid artery (ICA) until mild resistance was felt. After 90 min of occlusion, the filament was withdrawn to induce reperfusion. To confirm the proper occlusion of MCA, mice were monitored for regional cerebral blood flow (rCBF) with the Laser Doppler flowmetry (PeriFlux 5010; Perimed AB, Sweden) during and after surgery (Supplementary Fig. S1). A decline in rCBF ≥ 75% of baseline was considered as a successful occlusion. During the operation, body temperature was maintained at 37 ± 0.5 °C with a heating pad. The sham-operated mice underwent the same surgery except that the filament was not inserted into ICA.

### Cell culture and OGD model

Primary microglia cells were cultured as previously described^21^. Briefly, a mixed glia culture system was established and maintained in Dulbecco’s Modified Eagle’s Medium (DMEM, Gibco) with 10% fatal bovine serum (Gibco, USA) and 1% penicillin–streptomycin (Gibco, USA) for 14 days. Microglia cells were collected using a shaking method and seeded in plates for further use. To carry out OGD, cell culture medium was replaced with glucose-free DMEM (Gibco, USA); the culture dish was then transferred into a sealed chamber equipped with AnaeroPack-Anaero (Mitsubishi Gas Chemical Co., Inc., Japan). After 8 h, cells were returned to normal incubator with normal medium for reperfusion. The primary cortical neurons were prepared from C57BL/6J mouse brain (E16) and cultured in neurobasal medium (Gibco, USA) containing 2% B27 (Gibco, USA) and 1% GlutaMax (Gibco, USA). For microglia–neuron cocultures, Transwell® plates (0.4-μm pore size, Corning, MA, USA) were used. Primary neurons were seeded in the lower chamber of the Transwell plates and cultured together with microglia, which were pretreated with LP17 or control peptide and underwent OGD. The neurotoxicity was detected with the LIVE/DEAD™ Viability/Cytotoxicity Kit (Thermo Fisher Scientific, USA).

### Drug administration

TREM-1 inhibitory peptide LP17 (LQVTDSGLYRCVIYHPP) and control peptide (TDSRCVIGLYHPPLQVY) were chemically synthesized as previously reported (GenScript, China)^22^. LP17 (0.5 mg/kg or 1 mg/kg) was intranasally administered once daily for 3 consecutive days after MCAO. In vitro microglia were initially preincubated with LP17 (1 or 10 μM) for 2 h before OGD. After reoxygenation, microglia were then incubated with LP17 (1 or 10 μM) for another 24 h. To inhibit SYK, mice were injected intraperitoneally with R406 (5 mg/kg, Selleck, USA) once daily for 3 consecutive days after reperfusion. In vitro SYK inhibition experiments were conducted by pre-culturing microglia with 1 mM R406 before OGD, followed by 1 mM R406 for another 24 h after OGD. Vehicle animals received control peptide or normal saline instead. To detect cell proliferation, mice were received intraperitoneal injection of 5-bromo-2′-deoxyuridine (BrdU) (50 mg/kg, Sigma-Aldrich, USA) once daily for 7 consecutive days after MCAO surgery.

### Behavioral analysis

Modified Neurological Severity Scores (mNSS)^23^, an 18-point scoring system, was utilized to evaluate sensorimotor deficits 1 day before surgery and on day 3, 7, 14, and 28 post-surgery. To test long-term cognitive changes, Morris Water Maze (MWM) test was conducted from day 22 to day 28 post stroke as previous described^24^. Swimming paths were tracked and the average times to reach the submerged platform were recorded by the ANY-maze video-tracking software (Stoelting, USA).

### Magnetic resonance imaging (MRI) and infarct volume evaluation

MRI examinations were performed to determine infarct volume 3 days post-stroke using a 7.0 T MRI scanner (BRUKER PharmaScan, Germany). Briefly, mice were anesthetized with isoflurane and were placed on a nonmagnetic holder. Heart beat and respiration were continuously monitored. Rapid acquisition with relaxation enhancement-T2 sequence was used to acquire T2-weighted images with the following parameters: matrix = 256 × 256, field of view (FOV) = 20 mm × 20 mm, repetition time (TR) = 2800 ms, echo time (TE) = 50 ms, slice thickness = 0.5 mm (16 slices per animal). For diffusion weighted imaging (DWI), the images were acquired with a 256 × 256 matrix, FOV of 20 mm × 20 mm, TR of 5000 ms, TE of 22 ms, and slice thickness of 0.5 mm (16 slices per animal). The infarcts were identified by high signals acquired from T2-WI and further verified by DWI. Infarct volumes were calculated according to DWI scans as previous described^19^. Non-infarcted volume in the lesioned hemisphere (NVL) and the volume of the contralateral hemisphere (VC) were summed and multiplied by 0.5 mm. Then the relative infarct volume was calculated as (VC − NVL)/(2 × VC) × 100%.

### RNA-sequencing (RNA-seq) analysis

Total RNA of peri-infarct tissue from MCAO and sham-operated groups were extracted using TRIzol Reagent (Invitrogen, USA), and were quantified by NanoDrop (Thermo Fisher Scientific, USA). Then next generation sequencing library preparations were constructed using 1 μg of total RNA of each sample according to the manufacturer’s protocol (NEBNext® Ultra™ RNA Library Prep Kit for Illumina®, USA). RNA-seq analysis was performed on HiSeq X Ten instrument (Illumina, San Diego, CA, USA). Paired end (2 × 150 bp) of sequencing was done. The obtained data were underwent quality trimming to remove low quality bases using Cutadapt version 1.9.1^25^, followed by aligned to the mouse genome (Mus musculus. GRCm38) downloaded from Ensembl database (www.ensembl.org/info/data/ftp/) using HISAT2 version 2.0.1^26^. HTSeq version 0.6.1^27^ was used to quantify gene expression levels from the pair end clean data. Differential mRNA expression analysis was carried out using the DESeq2, version 1.6.3^28^. Adjusted *p* value of <0.05 was setted to detect differential expressed genes with at least two-fold change of expression.

### TREM-1 small interfering RNA (siRNA) transfection

Negative control siRNA (UCUCCGAACGUGUCACGUT) and TREM-1 siRNA (CCCAGTGACACAACTACAA) were synthesized by RIBOBIO (China). siRNA (20 μM) of 1.25 μl and riboFECT^TM^ CP Regent (RIBOBIO, China) of 3 μl were mixed in 30 μl riboFECT^TM^ CP Buffer to generate a transfection mixture. Then the mixture was added into DMEM (siRNA concentration: 50 nM) and cultured with microglia for 48 h. Real-time polymerase chain reaction (PCR) and western blot analysis were performed to examine the transfection efficiency.

### Histological staining

Mice were anesthetized and intracardially perfused with 0.9% sodium chloride followed by 4% paraformaldehyde (PFA). After postfixed in 4% PFA for 12 h, brains were dehydrated in gradient sucrose solutions of 10, 20, and 30% at 4 °C. Brains were then embedded in optimal cutting temperature compound (Sakura Finetek, USA) and cut into 15- or 25-μm sections.

#### Terminal deoxynucleotidyl transferase-mediated dUTP nick end labeling (TUNEL) staining

TUNEL staining was performed with the In Situ Cell Death Detection Kit, AP (Roche, USA) according to the manufacturer’s instructions. Briefly, frozen slides were sequentially incubated with 0.1% Triton X-100, 15% glacial acetic acid, and TUNEL mixture, followed by converter-AP. The signals were detected using BICP/NBT solution (Beyotime, China). Images were captured with an Olympus BX51 microscope.

#### Fluoro-Jade C (FJC) staining

Degenerated neurons were detected by FJC (Millipore, USA) as previously described^29^. Frozen sections were sequentially immersed in 1% NaOH/80% ethanol solution, 70% ethanol, and 0.06% potassium permanganate solution. The slides were then stained with 0.0001% FJC working solution.

#### Immunofluorescence staining

Samples were blocked with a solution containing 0.3% Triton, 3% goat serum, and 1% bovine serum albumin. To label BrdU, sections were preincubated with 2N HCL at 37 °C, followed by incubation with rat monoclonal antibody against BrdU (1:400; ab6326; Abcam, UK). For others, sections were incubated with antibody against TREM-1 (1:200; ab217161, Abcam, UK), Iba-1 (1:500; #019-19741, Wako, Japan), CD68 (1:200; MCA1957GA, AbD Serotec, UK), GFAP (1:500; ab4648, Abcam, UK), NeuN (1:500; ab177487, Abcam, UK), MBP (1:500; ab7349, Abcam, UK), vWF (1:500; ab6994, Abcam, UK), MPO (1:200; sc-16128-R, Santa Cruz Biotechnology, USA), SYK (1:200; #13198, Cell Signaling Technology, USA), and Gasdermin D (GSDMD) (1:200; sc-393581, Santa Cruz Biotechnology, UK) overnight at 4 °C. Sections were then incubated with appropriate secondary antibodies and DAPI (Sigma-Aldrich, USA). The positive signal was quantified by Image J software (NIH, USA).

### Enzyme-linked immunosorbent assay (ELISA)

Brain tissues from ischemic penumbra zone were dissected and homogenized. Cell culture supernatants were collected. The concentrations of IL-1β and IL-18 protein were detected utilizing the ELISA kits (Abcam, UK) according to the manufacturer’s instructions.

### Electron microscopy

Peri-infarct tissue (1 × 1 × 1 mm) samples were immersed in 2.5% glutaraldehyde and then fixed in 1% osmium tetroxide. After dehydrated in ethanol and embedded in araldite, tissues were cut into 50–60-nm sections. Finally, the thin sections were stained with 3% uranyl acetate and lead citrate, and were then scanned using H7500 Transmission Electron Microscope (Hitachi, Japan).

### Quantitative real-time PCR

Total RNA was extracted from peri-infarct tissues using TRIzol regent (Sigma-Aldrich, USA), and was reverse transcribed into cDNA with the RevertAid First Strand cDNA Synthesis Kit (Thermo Fisher Scientific, USA). Then quantitative real-time PCR was performed with UltraSYBR Mixture (CWBio, China), specific mouse primers (listed in Supplementary Table S1) and cDNA using the Mx3000P Real-Time PCR System (Agilent Technologies, USA). The mRNA expression of GAPDH was set as internal control. The results were expressed as fold changes compared with sham group in vivo or control group in vitro.

### Co-immunoprecipitation

Total cell lysates from cerebral tissues or primary microglia were extracted and prepared by using weak radioimmunoprecipitation assay (RIPA) lysis buffer (Beyotime, China). Protein extracts of 500 μg were incubated with 1 μg of antibody against TREM-1 or control IgG overnight at 4 °C. The immune complexes were then linked to protein A/G-agarose beads (Cell Signaling Technology, USA) for 4 h. Finally, the eluted proteins were collected and were analyzed by immunoblotting.

### Immunoblotting

Protein lysates were prepared from brain tissues and cultured microglia using RIPA lysis buffer (Cell Signaling Technology, USA) and were quantified by the BCA protein assay kit (Thermo Fisher Scientific, USA). Proteins of 30 μg were loaded and separated on 10% sodium dodecyl sulfate polyacrylamide gel electrophoresis gels and then transferred to polyvinylidene difluoride membranes (Millipore, USA). Membranes were incubated with primary antibodies against TREM-1 (1:1000; ab217161, Abcam, UK), MPO (1:500; sc-16128-R, Santa Cruz Biotechnology, USA), intracellular adhesion molecule-1 (ICAM-1) (1:500; sc-1511, Santa Cruz Biotechnology, USA), SYK (1:1000; #13198, Cell Signaling Technology, USA), phosphorylated-SYK (p-SYK; 1:1000; ab195732, Abcam, UK), CARD9 (1:1000; ab124922, Abcam, UK), NLRP3 (1:500; sc-66846, Santa Cruz Biotechnology, USA), ASC (1:500; sc-33958, Santa Cruz Biotechnology, USA), caspase-1 p10 (1:500; sc-514, Santa Cruz Biotechnology, USA), IL-1β (1:500; sc-7884, Santa Cruz Biotechnology, USA), IL-18 (1:1000; ab71495, Abcam, UK), NF-κB p65 (1:1000; #8242, Cell Signaling Technology, USA), phosphorylated-NF-κB p65 (Ser536) (1:1000; #3033, Cell Signaling Technology, USA), GSDMD (1:500; sc-393581, Santa Cruz Biotechnology, USA), postsynaptic density protein 95 (PSD95; 1:1000; ab18258, Abcam, UK), calcium/calmodulin-dependent protein kinase II (CaMKII; 1:1000; ab52476, Abcam, UK), Synapsin I (1:1000; ab18814, Abcam, UK), Synaptophysin (1:1000; ab32127, Abcam, UK), and β-actin (1:1000; #4970, Cell Signaling Technology, USA) overnight at 4 °C, followed by incubated with HRP-conjugated secondary antibody at room temperature. The protein signals were detected by Immobilon Western Chemiluminescent HRP substrate (Millipore, USA) and were quantified by Image J software (NIH, USA). The expression of β-actin was set as internal control.

### Statistical analysis

SPSS 22.0 software (IBM, Armonk, NY, USA) was used for data analysis. Differences between groups were compared using two-tailed Student’s *t* tests and one-way ANOVA followed by Tukey’s post hoc test. Escape latency and swimming path length in MWM test were analyzed by two-way repeated-measures ANOVA followed by Tukey’s post hoc test. All data are expressed as mean ± SEM. Statistical significance was determined as *p* < 0.05.

## Results

### Transcriptional regulation of immune genes in peri-infarct area

**Fig. 1:**
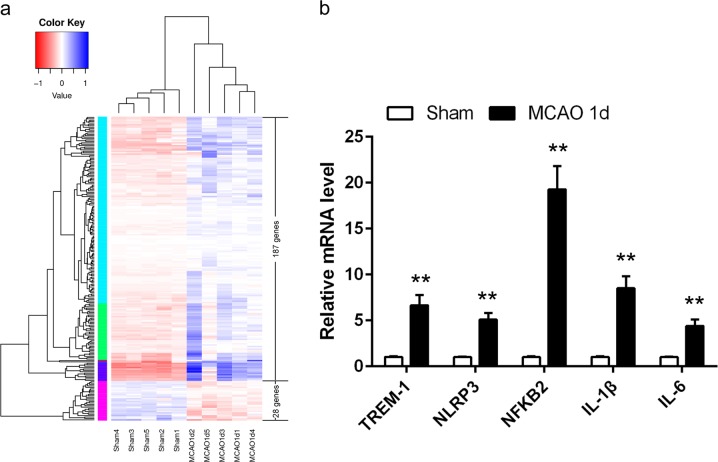
Focal cerebral ischemia-induced immune gene regulation in peri-infarct area 1 day post-modeling. **a** Heatmap of the immune genes with an at least two-fold change in expression in cerebral peri-infarct tissues compared with the levels in sham samples. **b** Real-time PCR analysis of TREM-1, NLRP3, NFKB2, IL-1β, and IL-6. Data are expressed as mean ± SEM, *n* = 5 in each group. **p *<* 0.01 vs. sham group

To explore neuroinflammatory response following ischemic insult, we employed RNA-seq analysis to detect the expression of 215 genes, which may participate in the immune process (Supplementary Table S2). Among these immune genes, 187 were upregulated and 28 were downregulated in the MCAO mice compared with the sham-operated mice 1 day after MCAO surgery (Fig. 1a). It is worthy to note that TREM-1 and NLRP3 inflammasome related genes (NLRP3, NFKB2, IL-1β, and IL-6) were significantly elevated (Supplementary Table S2). To further confirm these results, real-time PCR was performed. As shown in Fig. 1b, there were substantial increases in the mRNA levels of these inflammatory factors following cerebral ischemia (all *p* < 0.01).

### The expression pattern and time course of TREM-1 expression under ischemic–hypoxic condition

**Fig. 2:**
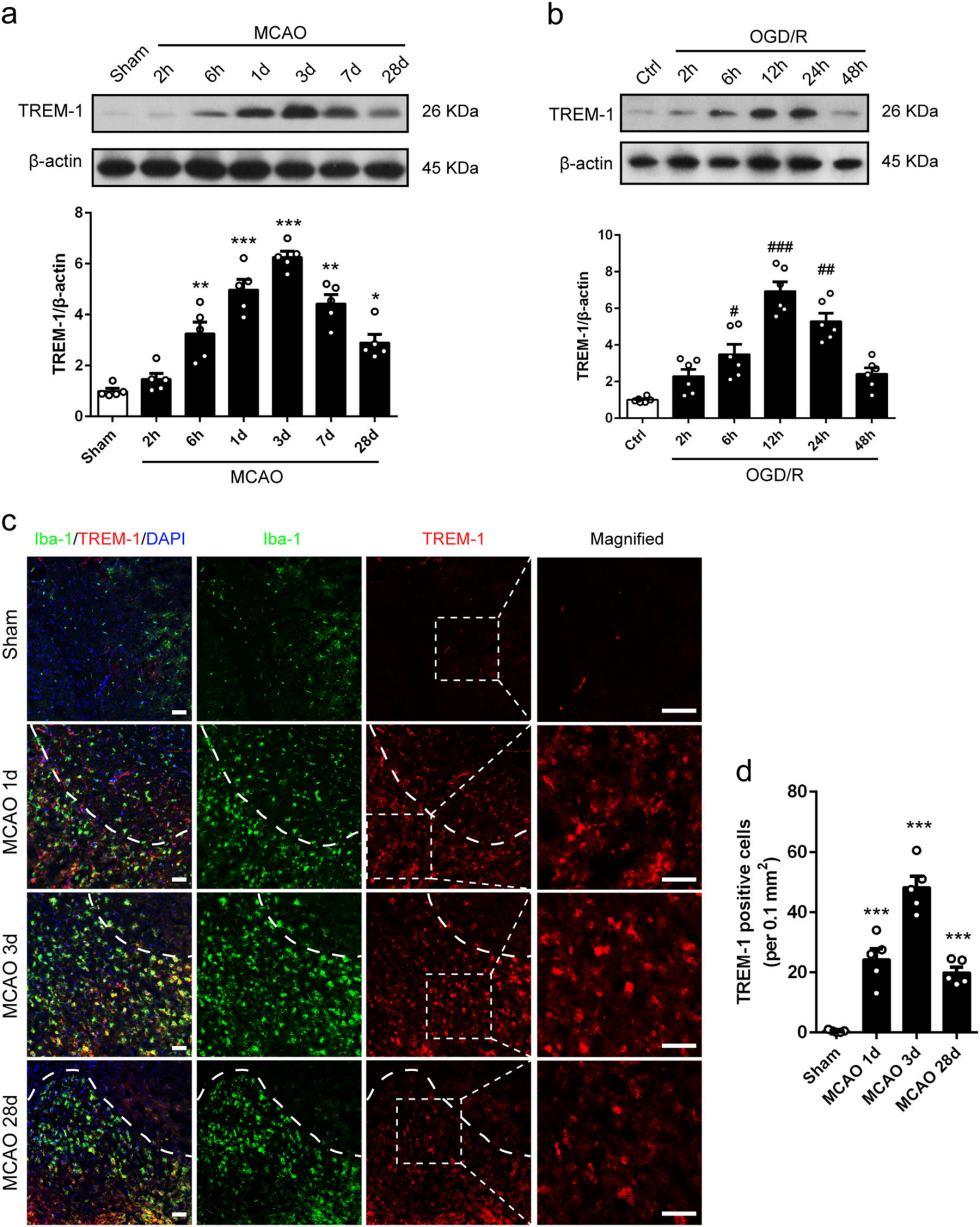
TREM-1 in microglia was upregulated post-stroke. **a** Immunoblots and quantitative analysis of TREM-1 at 2 and 6 h, and 1, 3, 7, and 28 days post-stroke. *n* = 5. **b** Immunoblots and quantitative analysis of TREM-1 at 2, 6, 12, 24, and 48 h after reoxygenation. *n* = 6. **c** Co-staining for TREM-1 (red) and Iba-1 (green) at 1, 3, and 28 days after reperfusion in peri-infarct zone. Magnified views of TREM-1 staining are marked with dashed line boxes. **d** Quantification of TREM-1 positive cells. *n* = 5. Data are expressed as mean ± SEM; *p *<* 0.05, **p *<* 0.01, ***p *<* 0.001 vs. sham group; ^#^*p* < 0.05, ^##^*p* < 0.01, ^###^*p* < 0.001 vs. control group. Scale bar = 50 μm

We next explored the protein expression pattern and time course of TREM-1 in MCAO mice and OGD microglia. Western blotting results demonstrated that TREM-1 was marginally increased at 6 h after surgery, peaked at 3 days after reperfusion, and gradually declined (Fig. 2a). The protein level of TREM-1 remained significantly higher at 28 days post-stroke (Fig. 2a). Similarly, OGD increased microglial TREM-1 expression in a time depend manner, with a peak expression at 12 h after reoxygenation (Fig. 2b). Consistently, robust TREM-1 positive cells co-stained with Iba-1 were detected 3 days post-stroke in peri-infarct zone, indicating a microglial autonomous increment of TREM-1 (Fig. 2c, d, *p* < 0.001). However, TREM-1 staining signal was not detected on astrocyte, neuron, oligodendrocyte, and endothelial cell (Supplementary Fig. S2).

### Suppression of TREM-1 ameliorates infarct progression and neuronal injury

**Fig. 3:**
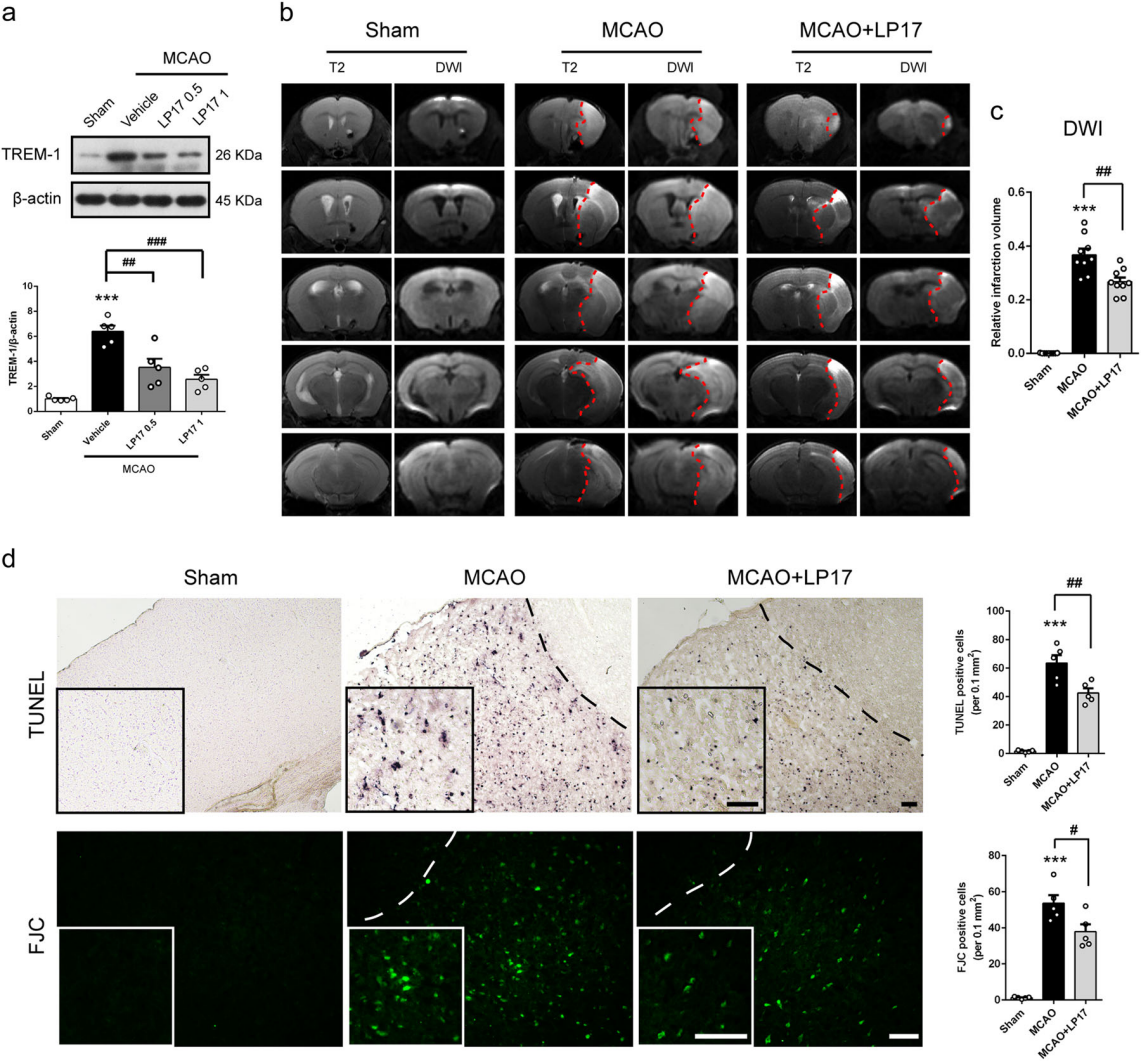
TREM-1 inhibition decreased ischemic infarction and rescued neuronal damage 3 days after reperfusion. **a** Protein levels of TREM-1 in peri-infarct tissues after treating with LP17. *n* = 5. **b** Representative images of five different coronal slices in T2 and DWI MRI scans. **c** Quantification of infarct volume according to DWI scans. *n* = 9. **d** TUNEL and FJC staining showed that the density of TUNEL- and FJC-positive cells were increased after I/R, while LP17 treatment decreased the positive signals in the peri-infarct area. Insets show a higher magnification view. *n* = 5. Scale bar = 50 μm. Data are expressed as mean ± SEM; ***p *<* 0.01 vs. sham group; ^#^*p* < 0.05, ^##^*p* < 0.01, ^###^*p* < 0.001 vs. MCAO group

To confirm the biological function of TREM-1 in ischemic stroke, we employed intranasal delivery of an inhibitory peptide called LP17 to block TREM-1 expression. LP17 was labeled with rhodamine to determine whether it could get access into brain. As shown in Supplementary Fig. S3, red fluorescent signals of LP17 were detected in olfactory bulb, cortex, and hippocampus after intranasal administration. Ischemia-induced TREM-1 elevation was strikingly abolished under LP17 of 1 mg/kg (Fig. 3a, *p* = 0.0001). These results indicated that LP17 could get access into brain and block TREM-1. To detect the effects of LP17 on infarct volume post-stroke, T2-weighted image and DWI were performed. LP17 administration significantly reduced infarct volume by 27.3% compared to MCAO mice receiving control peptide (Fig. 3b, c, *p* = 0.002). We next asked whether LP17 could rescue ischemia-induced neuronal injury. To this end, the TUNEL and the FJC assay were conducted to assess apoptosis and neuronal degeneration. As illustrated in Fig. 3d, supplementation of 1 mg/kg LP17 induced a markedly reduction in TUNEL positive cells and FJC positive neurons (*p* = 0.005 and 0.021).

### Pharmacological blockade of TREM-1 improves long-term neurobehavioral function

**Fig. 4:**
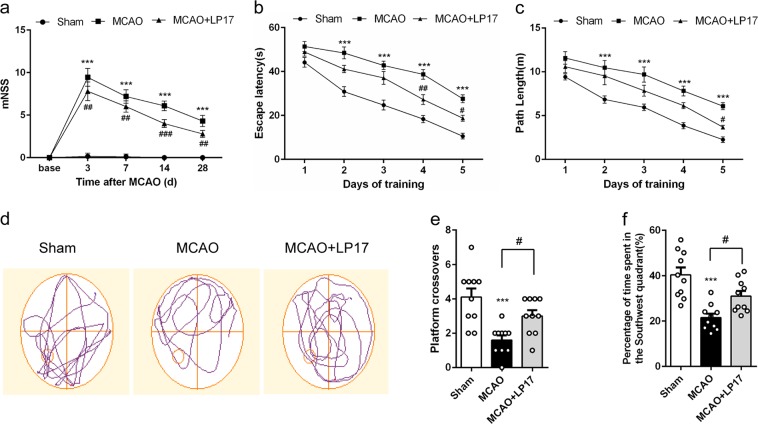
LP17 administration rescued neurological deficits and cognitive dysfunction of MCAO mice. **a** The mNSS of sham, MCAO, and MCAO + LP17 group. Mice were tested before MCAO surgery. MCAO 3 days: *n* = 20; other time points: *n* = 10. The escape latency (**b**) and path length (**c**) in the hidden platform phase. *n* = 10. **d** Representative swimming trajectories of the three groups in probe trials. The crossovers of the platform location (**e**) and the percentage of time spent in the target quadrant (**f**) were recorded and analyzed. *n* = 10. Data are expressed as mean ± SEM. ***p < 0.001 vs. sham group; ^#^*p* < 0.05, ^##^*p* < 0.01, ^###^*p* < 0.001 vs. MCAO group

Post-stroke behavioral dysfunction has been reported to be associated with neuroinflammation^30^. First, neurological function was evaluated by mNSS. MCAO-treated mice exhibited sustained sensorimotor deficits, which can be significantly reversed by LP17 treatment (Fig. 4a). We then tested long-term spatial learning and memory by MWM. No differences were noted in escape latency and swimming speed in cued trials, suggesting that MCAO mice have no visual or swimming deficiency (Supplementary Fig. S4). Post hoc analysis showed that the escape latency and path length to reach the hidden platform of MCAO mice were inferior to that of sham-operated mice on days 2–5 of training. However, mice with TREM-1 inhibition demonstrated a better performance of reduced escape latency and shortened path length (Fig. 4b, c). Probe trails were carried out 24 h after the last training trial. The LP17-treated mice revealed more crossovers and spent more time in the target quadrant (Fig. 4d–f, *p* = 0.039 and 0.030).

**Fig. 5:**
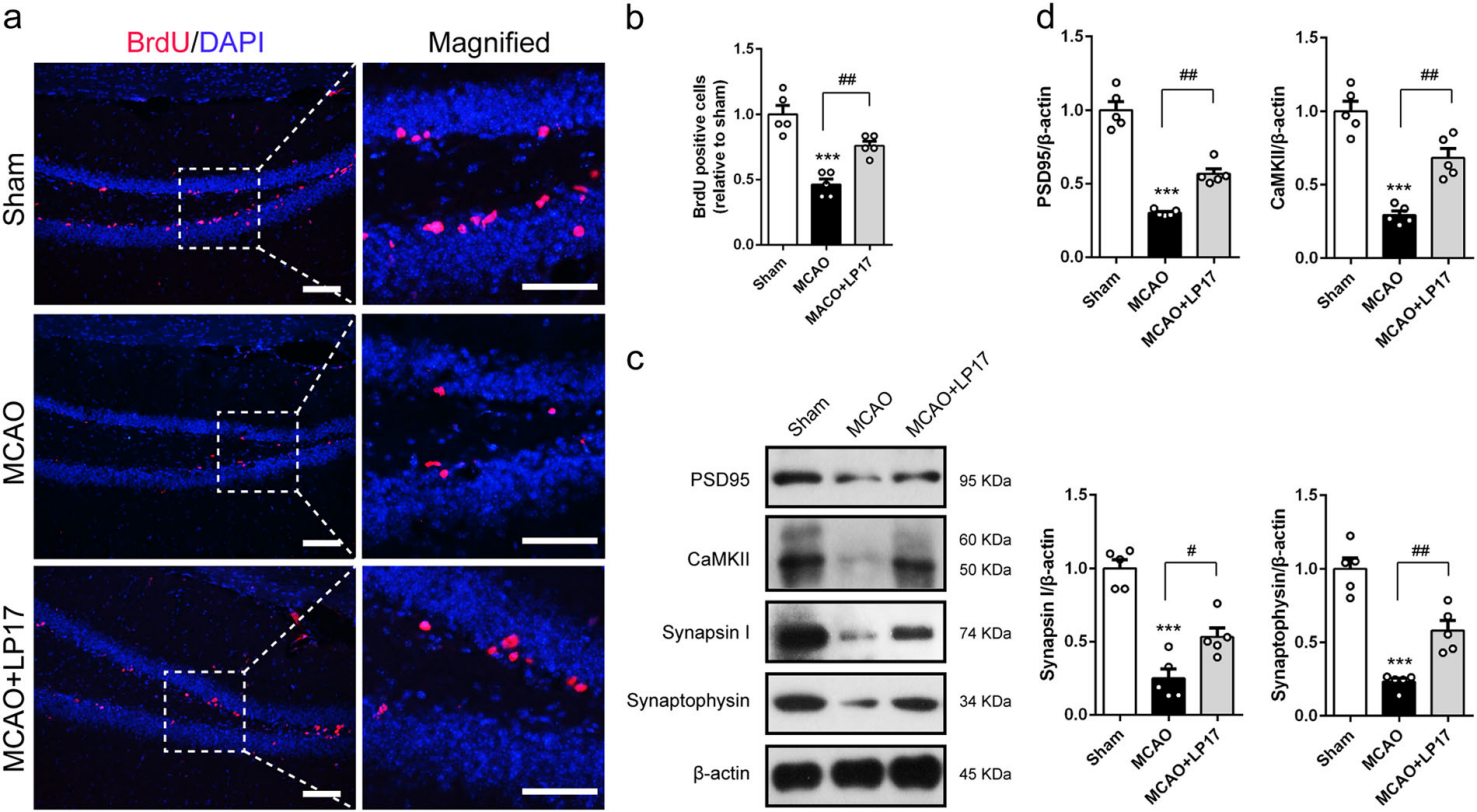
TREM-1 inhibition potentiated cell proliferation and neural plasticity in hippocampus following ischemic stroke. **a** Representative micrographs of BrdU-positive cells in dentate gyrus. Magnified views of BrdU staining are marked with dashed line boxes. Scale bar = 20 μm. **b** Quantitative analysis of BrdU-positive cells. **c**, **d** Western blots and quantification of synaptic elements, including PSD95, CAMKII, Synapsin I, and Synaptophysin. *n* = 5 in each group. Data are expressed as mean ± SEM; ***p < 0.001 vs. sham group; ^#^*p* < 0.05, ^##^*p* < 0.01 vs. MCAO group

Then we explored the underlying mechanisms involved in the functional improvement induced by LP17 supplementation. BrdU administration was used to mark cells in proliferation. The number of BrdU-positive cells in dentate gyrus was maintained at a low level at 28 days after MCAO, which was restored by administration of LP17 (Fig. 5a, b, *p* = 0.003). Neuroplasticity is another compensatory mechanism for brain rehabilitation after injury. We therefore assessed the expressions of synaptic proteins, including PSD95, CaMKII, Synapsin I and Synaptophysin in hippocampus 28 days post-MCAO. Inhibition of TREM-1 was associated with substantial high levels of all these proteins (Fig. 5c, d, *p* = 0.001, 0.009, 0.018, and 0.002). Overall, these data suggested that blockade of TREM-1 rescues long-term neurobehavioral impairment through promoting cell proliferation and synaptic plasticity in hippocampus following ischemic stroke.

### Suppression of TREM-1 inhibits microglial M1 polarization and neutrophil infiltration

**Fig. 6:**
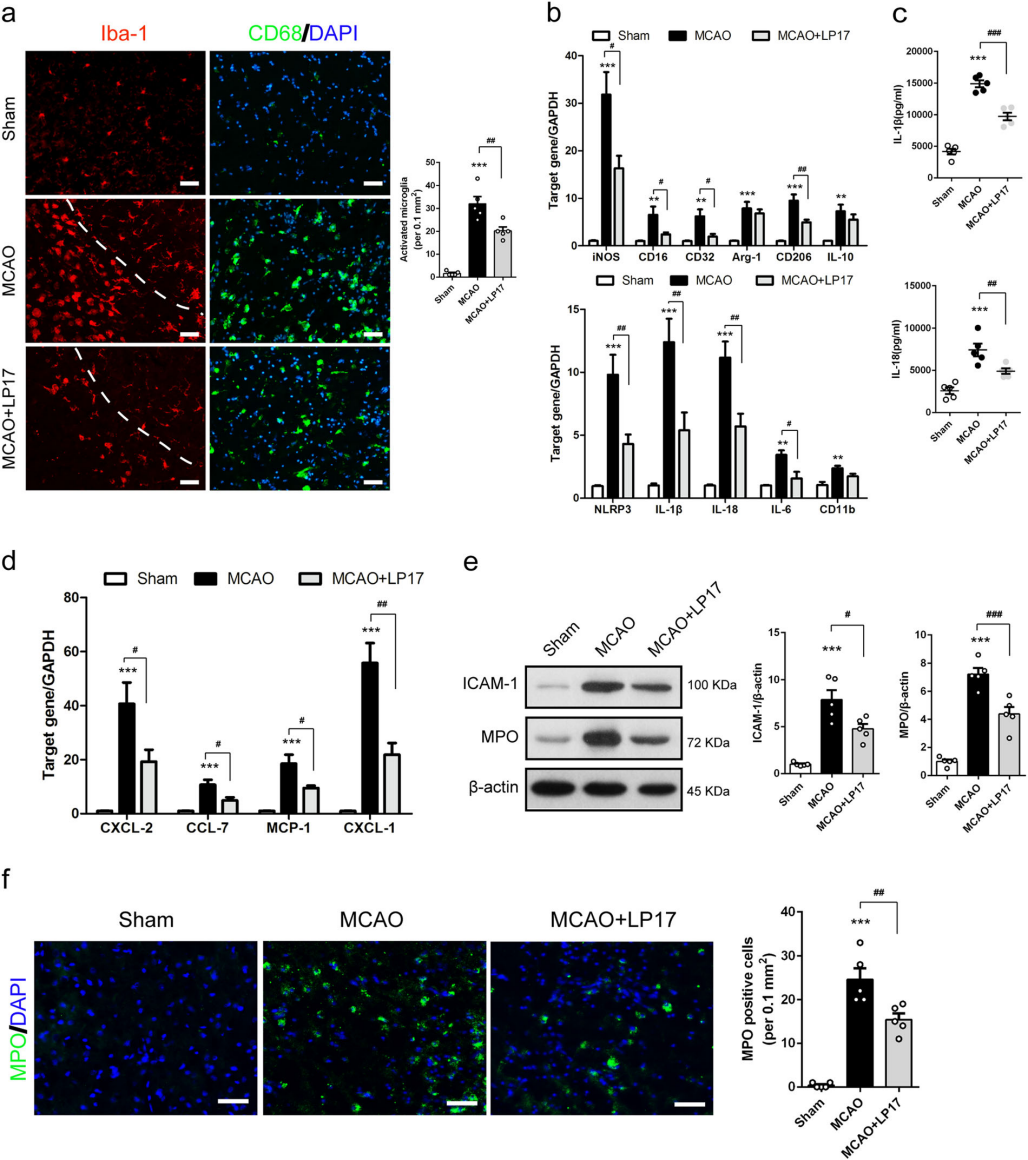
Blockade of TREM-1 suppressed inflammatory mediators and chemokines production and neutrophils invasion. **a** Immunostaining for Iba-1 and CD68 in sham, MCAO, and MCAO + LP17 group. **b** Relative mRNA expression of inflammatory genes (iNOS, CD16, CD32, CD11b, NLRP3, IL-1β, IL-18, and IL-6) and anti-inflammatory genes (CD206, Arg-1, and IL-10). **c** The ELISA assay for IL-1β and IL-18 in peri-infarct brain tissues. **d** Real-time PCR analysis for chemokines (CXCL-2, CCL-7, MCP-1, and CXCL-1). **e** Western blots showing expressions of ICAM-1 and MPO in the peri-infarct cortex with quantification. **f** MPO immunostaining among the three groups with quantification. *n* = 5 in each group. Data are expressed as mean ± SEM; **p < 0.01, ***p < 0.001 vs. sham group; ^#^*p* < 0.05, ^##^*p* < 0.01, ^###^*p* < 0.001 vs. MCAO group. Scale bar = 50 µm

Given the essential role of TREM-1 in innate immune system regulation^7^, we next determined whether inhibition of TREM-1 could affect post-ischemic inflammation. Iba-1 and CD68 staining were used to examine the response of microglia in peri-infarct region. Resting microglial cells in the sham group were morphologically noted with small soma and thin protrusions in Iba-1 staining (Fig. 6a). In control peptide-treated MCAO mice, activated microglia with bigger soma, shorter protrusions and amoeboid morphology were observed, while fewer activated microglia were detected in LP17-treated mice (Fig. 6a). CD68 staining further confirmed LP17-induced alleviation of microglial activation (Fig. 6a). Next, we sought to examine microglial polarization in peri-infarct region. mRNA levels of M1 markers, including iNOS, CD16, CD32, NLRP3, IL-1β, IL-18, IL-6, and CD11b, were all markedly increased 3 days after MCAO, but these increments, except for CD11b, were significantly attenuated by treatment with LP17 (Fig. 6b, *p* = 0.013, 0.041, 0.018, 0.007, 0.010, 0.004, 0.012, and 0.168). Among the M2 type genes, embracing Arg-1, CD206, and IL-10, only CD206 was suppressed after LP17 administration (Fig. 6b, *p* = 0.007). The ELISA assay revealed that intracellular upregulation of IL-1β and IL-18 mRNAs was accompanied by a parallel increment of extracellular protein levels of IL-1β and IL-18 in the MCAO group, which were both reversed by LP17 application (Fig. 6c).

We then set out to test whether TREM-1 inhibition influences chemokines production and neutrophils infiltration under cerebral ischemic injury. Peri-infarct brain tissue was subjected to real-time PCR for chemokines mRNA assessment. Pharmacological inhibition of TREM-1 induced remarkable reductions in mRNA levels of CXCL-2 (by 52.6%), CCL-7 (by 54.6%), MCP-1 (by 48.8%), and CXCL-1 (by 60.8%) (Fig. 6d, *p* = 0.040, 0.020, 0.025, and 0.001) following ischemic attack. Meanwhile, LP17 led to a 39.5% decrease in ICAM-1 expression (Fig. 6e, *p* = 0.015). Immunoblotting and immunostaining of MPO both provided direct evidences that LP17 treatment can mitigate neutrophils infiltration into the peri-infarct region (Fig. 6e, f, *p* = 0.0002 and 0.006). These results demonstrated that LP17 may dampen leukocytes infiltration.

### Inhibition of TREM-1 retards microglial sterile inflammation and neuronal death

**Fig. 7:**
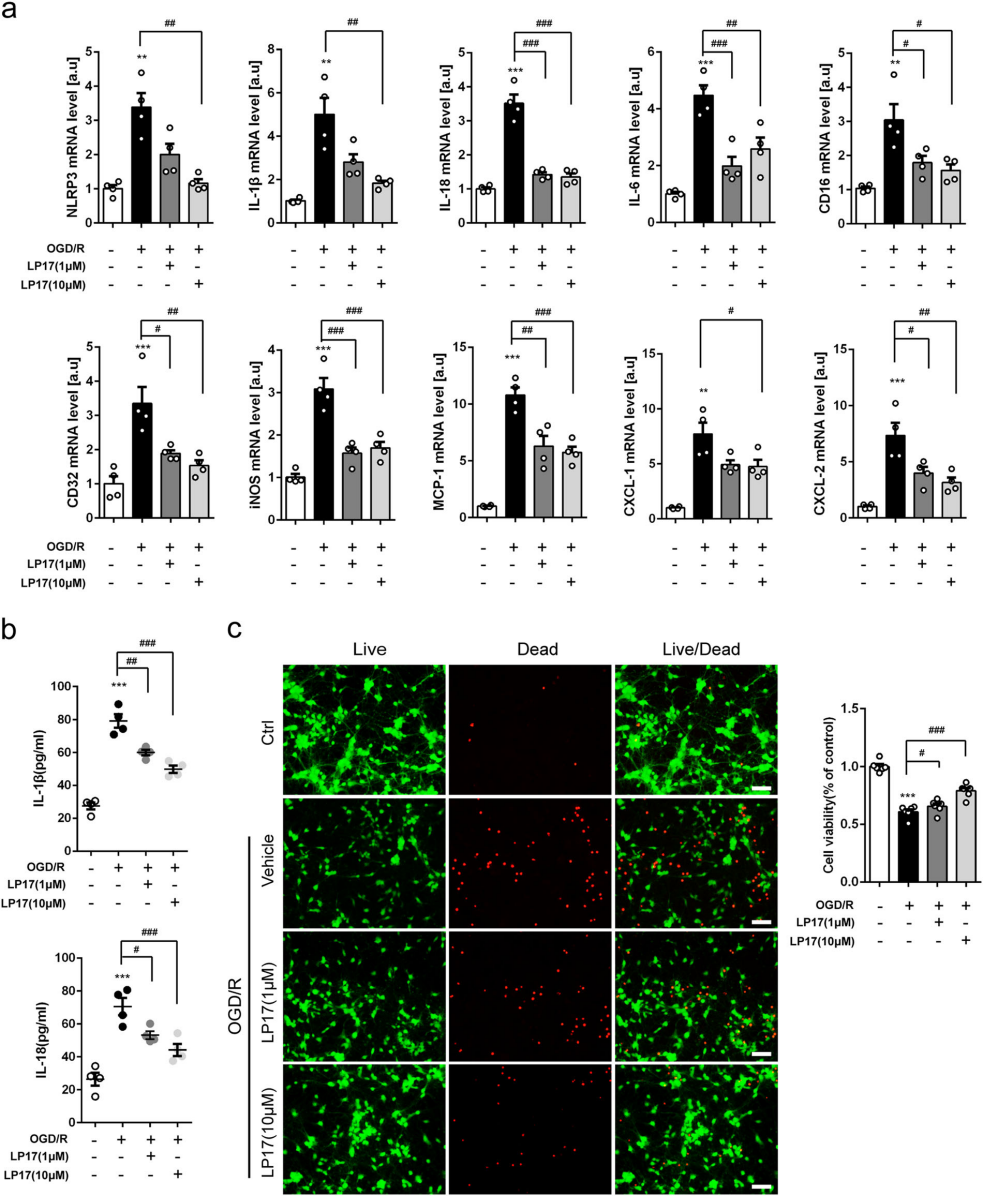
Pharmacological inhibition of TREM-1 suppressed inflammatory factors production and neuronal damage in vitro. **a** The mRNA levels of NLRP3, IL-1β, IL-18, IL-6, CD16, CD32, iNOS, MCP-1, CXCL-1, and CXCL-2 were analyzed by quantitative real-time PCR in primary microglia 24 h post-OGD. *n* = 4. **b** The ELISA assay for IL-1β and IL-18 in the supernatant of primary microglia 24 h after OGD. *n* = 4. **c** Neurons were co-cultured with control microglia, OGD microglia, or OGD microglia treated with LP17 (1 or 10 μM) for 24 h. Neuronal viability of the four groups was measured. *n* = 6. Scale bar = 50 µm. Data are expressed as mean ± SEM. **p < 0.01, ***p < 0.001 vs. control group; ^#^*p* < 0.05, ^##^*p* < 0.01, ^###^*p* < 0.001 vs. OGD/R group

We further studied TREM-1 function in the microglial OGD model. Different doses of LP17 (1 or 10 μM) were added. By real-time PCR, 10 μM LP17 substantially decreased mRNA levels of pro-inflammatory cytokines (NLRP3, IL-1β, IL-18, IL-6, CD16, CD32, and iNOS) and chemokines (MCP-1, CXCL-1, and CXCL-2) 24 h after reoxygenation (Fig. 7a). LP17 remarkably attenuated extracellular protein levels of IL-1β and IL-18, with maximal reduction observed at 10 μM (Fig. 7b). To determine whether inhibition of microglial TREM-1 affects neuronal survival, a microglia–neuron coculture system was introduced. Neurons were co-cultured with OGD microglia in the absence or presence of LP17 for 24 h. OGD induced substantial increment of neuronal death as determined by quantification of live/dead staining, while incubation with LP17 maximally preserved neuronal viability under the concentration of 10 μM (Fig. 7c, *p* < 0.001).

### TREM-1 interacts with microglial SYK

**Fig. 8:**
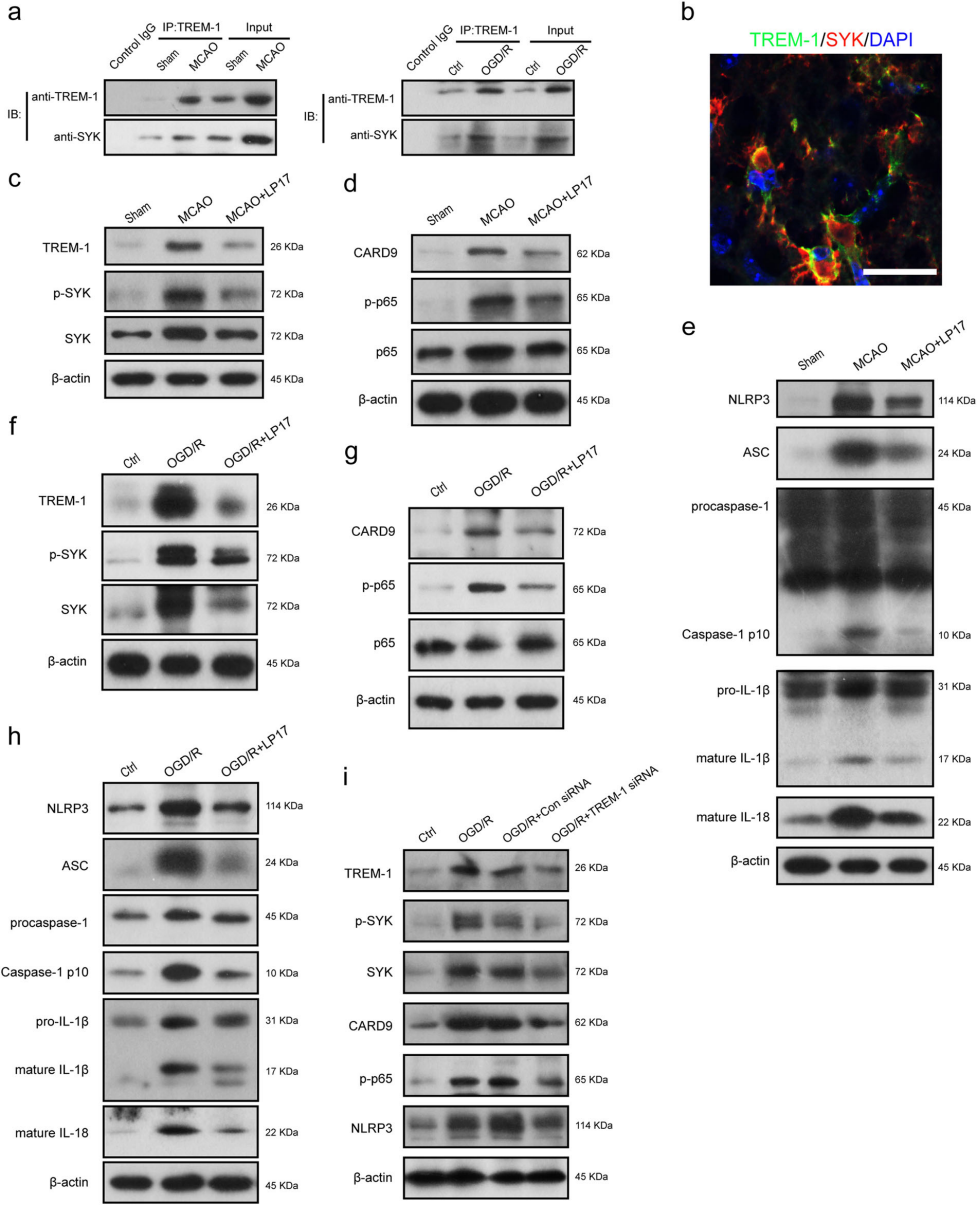
TREM-1 triggered the CARD9/NF-κB signaling pathway and NLRP3 inflammasome through interacting with SYK. **a** The lysates from cortex tissue and microglia were immunoprecipitated with anti-TREM-1. Then immunoprecipitates were analyzed by immunoblotting with anti-TREM-1 and anti-SYK. **b** Immunostaining for TREM-1 and SYK in peri-infarct area. Scale bar = 20 μm. **c** TREM-1, p-SYK, and SYK protein expressions in ischemic penumbra tissues were detected by western blotting 3 days after reperfusion with or without LP17 administration. **d** Immunoblotting for the CARD9/NF-κB signaling pathway using the same tissues in **c**. **e** Immunoblotting analysis for NLRP3, ASC, caspase-1, IL-1β, and IL-18. **f** OGD microglia cells were treated with LP17 (10 μM). Protein levels of TREM-1, p-SYK, and SYK were determined by western blots 24 h after reoxygenation. **g**, **h** Cell lysates with the same treatments in **f** were subjected to western blots for detecting CARD9, p-p65, p65, NLRP3, ASC, caspase-1, IL-1β, and IL-18. **i** Primary microglia cells were treated with Con siRNA or TREM-1 siRNA, and then were subjected to OGD for 8 h. Protein levels of TREM-1, p-SYK, SYK, CARD9, p-p65, and NLRP3 were detected by immunoblotting 24 h after reoxygenation. *n* = 5 in each group

It has been reported that engagement of TREM-1 leads to recruitment and stimulation of kinase SYK in monocytes/macrophages, delivering an activation signal to downstream pathways^7^. We examined whether an association exists between TREM-1 and SYK in microglia. By co-immunoprecipitation, TREM-1 appeared to interact with SYK both in vivo and in cultured microglia (Fig. 8a). Co-immunolabeling further revealed that TREM-1 receptor was co-localized with microglial SYK, rendering a physical basis for their interaction (Fig. 8b).

### TREM-1 is necessary for ischemia-induced activation of SYK and downstream components

To depict whether TREM-1 can mobilize SYK, the expressions of molecules in downstream CARD9/NF-κB and NLRP3/caspase-1 pathways were estimated^31^. LP17 and TREM-1 siRNA were both introduced. In vivo, I/R-increased TREM-1 expression was accompanied by the elevation of SYK and p-SYK after reperfusion (Fig. 8c, Supplementary Fig. S5a). I/R also induced noticeable increases in CARD9, p-p65 in CARD9/NF-κB signaling and NLRP3, ASC, cleaved caspase-1, mature IL-1β, and mature IL-18 in NLRP3/caspase-1 signaling (Fig. 8d, e, Supplementary Fig. S5b, c). These increments were all suppressed by LP17, suggesting that TREM-1 could activate SYK following MCAO; and this activation of SYK could lead to increased protein levels of components in CARD9/NF-κB and NLRP3/caspase-1 pathways. On the other hand, in vitro, we found a consistent effect of LP17 on SYK initiation and downstream signals activation (Fig. 8f–h, Supplementary Fig. S5d–f). Besides, cultured microglia cells were incubated with TREM-1 siRNA, which can effectively knock down TREM-1 expression. We found that OGD-induced upregulation of TREM-1, p-SYK, SYK, CARD9, p-p65, and NLRP3 were markedly suppressed by TREM-1 siRNA (Fig. 8i, Supplementary Fig. S5g). This counteraction was comparable to the function of LP17. Taken together, these data suggest that TREM-1 is necessary in SYK mobilization and downstream CARD9/NF-κB and NLRP3/caspase-1 activation in microglia after ischemic injury.

### TREM-1-induced SYK mobilization is responsible for microglial pyroptosis

**Fig. 9:**
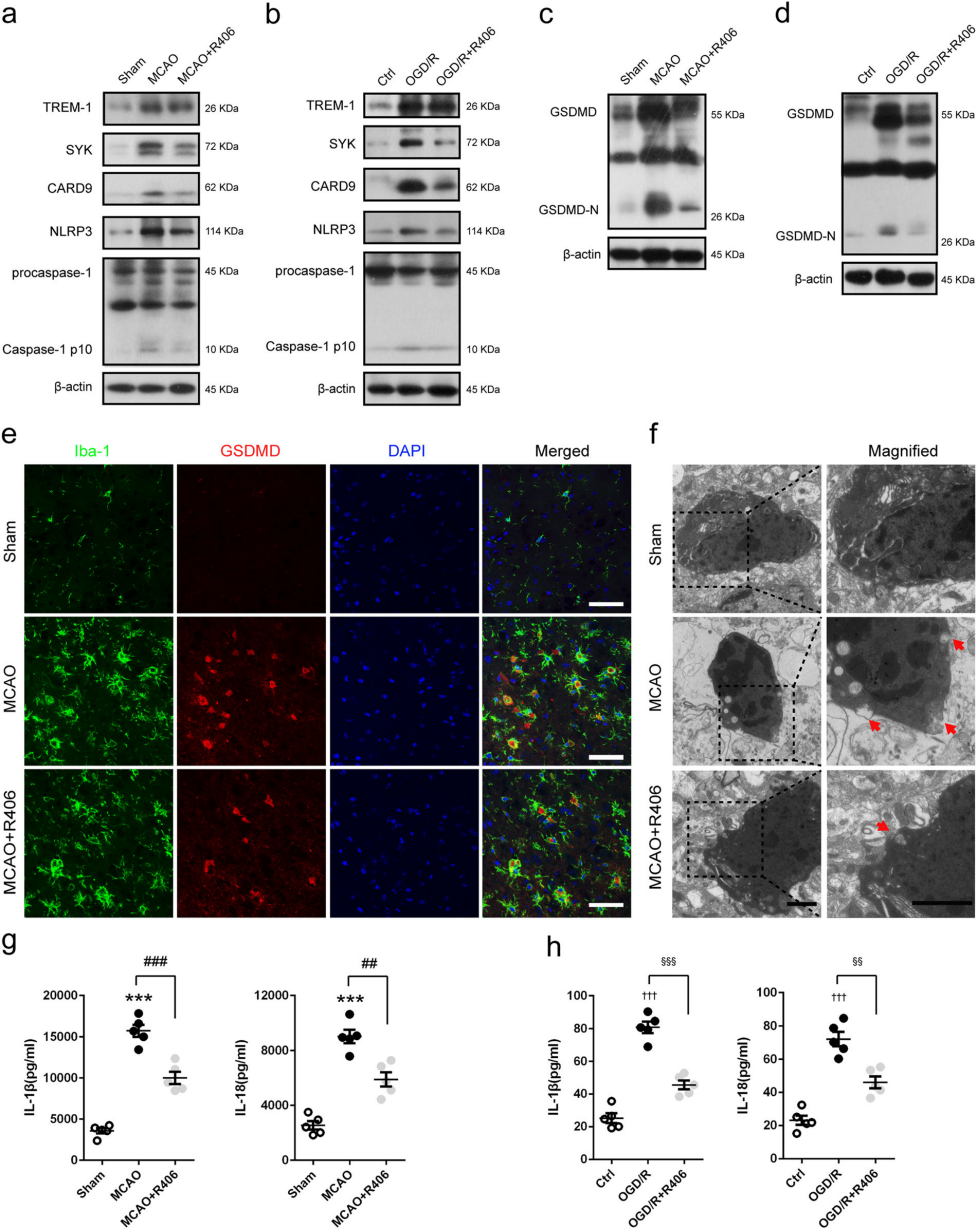
TREM-1-induced SYK activation triggered microglial pyroptosis post-stroke. MCAO mice and OGD microglia were treated with SYK inhibitor, R406. **a**, **b** Western blotting analysis of TREM-1, SYK, CARD9, NLRP3, and caspase-1 in MCAO mice 3 days after reperfusion and in OGD microglia 24 h after reoxygenation. **c**, **d** Immunoblotting analysis for GSDMD in treated mice and cultured microglia. **e** Double immunostaining of Iba-1 and GSDMD revealed a good co-localization of these two makers. Treatment with LP17 reduced GSDMD positive microglia in ischemic penumbra. Scale bar = 50 µm. **f** Representative transmission electron microscopy images of microglia in peri-infarct area. Magnified views of microglial cytomembrane are marked with dashed line boxes. Red arrow head: membrance pores. Scale bar = 1 µm. **g**, **h** ELISA assays for IL-1β and IL-18 in brain tissues and microglial culture medium. *n* = 5 in each group. Data are expressed as mean ± SEM. ***p < 0.001 vs. sham group; ^##^*p* < 0.01, ^###^*p* < 0.001 vs. MCAO group; ^†††^*p* < 0.001 vs. control group; ^§§^*p* < 0.01, ^§§§^*p* < 0.001 vs. OGD/R group

Caspase-1 activated by SYK can cleave GSDMD to trigger pyroptosis^32^. Pyroptosis is a lytic type of programmed cell death that inherently results in inflammation^32^. Thus, microglial pyroptosis may be an important contributor to exacerbate post-stroke neuroinflammation. To this end, we treated mice or microglial cells with R406 to inhibit SYK. Without noticeable disturbance of the elevated TREM-1 level, R406 administration can significantly reverse the increased immunoreactivities of SYK, CARD9, NLRP3, and caspase-1 p10 in MCAO mice and OGD/R microglia (Fig. 9a, b, Supplementary Fig. S6a, b). We then gauged pyroptosis after SYK inhibition both in vivo and in vitro. Immunoblotting results showed that GSDMD-N, a cleaved form of increased GSDMD, displayed a 7.60-fold and 5.76-fold higher expression following MCAO and OGD/R; while R406 treatment markedly downregulated ischemia-induced elevation of GSDMD and GSDMD-N both in vivo (by 42.8% and 38.7%) and in vitro (by 43.9% and 44.7%) (Fig. 9c, d, Supplementary Fig. S6c, d). Consistently, double immunofluorescence results implied that more GSDMD positive microglial cells were detected in the peri-infarct region of MCAO mice 3 days post-stroke compared to that in sham-operated mice, which were remarkably attenuated by R406 (Fig. 9e, Supplementary Fig. S7). Transmission electron microscope was employed to detect pores formed by GSDMD-N on microglia membrane. As manifested in Fig. 9f, membrane pores were less frequent in R406-treated MCAO mice compared to vehicle-treated MCAO mice. The ELISA assay demonstrated that extracellular expressions of IL-1β and IL-18 were both refrained by R406 (Fig. 9g, h, *p* = 0.0001, 0.0011 and *p* = 0.0003, 0.002).

## Discussion

Ischemic cerebral injuries lead to permanent damages to the brain lacking blood flow, whose progression involve an essential step of post-ischemic inflammation, and may affect physical functioning and cognition. Concomitantly with previous studies^33^, a robust inflammatory response was detected in our experimental ischemic models, as reflected by enhanced microglial M1 polarization and neutrophil recruitment. M1 phenotype microglia could exert cytotoxic effects by producing inflammatory cytokines, reactive oxygen, and nitrogen species^34,35^, while infiltrated neutrophils could exaggerate blood brain barrier (BBB) damage through exacerbating oxidative stress and inducing blood flow obstruction^36,37^. In the present study, we found that these inflammatory reactions were accompanied by increased infarct volume, neuronal injury, and long-term functional impairment.

TREM-1 was initially detected in peripheral blood monocytes and neutrophils, and subsequently was found in macrophages, endothelial cells, and fibrosarcoma cell line^10^. Our double immunostaining results showed that TREM-1 was localized on microglia in peri-infarct area. Microglial TREM-1, a known receptor in regulating cerebral immune reactions^38^, was robustly induced in a time-dependent manner with the peak at 3 days post-stroke. In our study, we found that blockade of TREM-1 could not attenuate the increased mRNA level of CD11b in MCAO mice. We think the complex microenvironment in brain may be responsible for this phenomenon. Consistent with our result, Hommes et al.^39^ did not find that TREM-1 ablation could affect cellular CD11b expression. Although LP17 treatment did not reduce the expression of CD11b, it did decrease the expression of other M1 markers, suggesting that TREM-1 activation could promote microglial M1 polarization. Similar phenomenon has been described during oligoarticular juvenile idiopatic arthritis in which TREM-1 engagement induced macrophage M1 pro-inflammatory reprogramming^40^. It is reported that TREM-1 stimulation can induce the generation of inflammatory mediators, MPO release, and the upregulation of adhesion molecules^41^. Consistently, we found that ischemia-induced TREM-1 promoted IL-1β, IL-18, IL-6, CXCL-2, MCP-1, and CXCL-1 productions in microglia as well as the expression of MPO and ICAM-1 following ischemic injury.

**Fig. 10:**
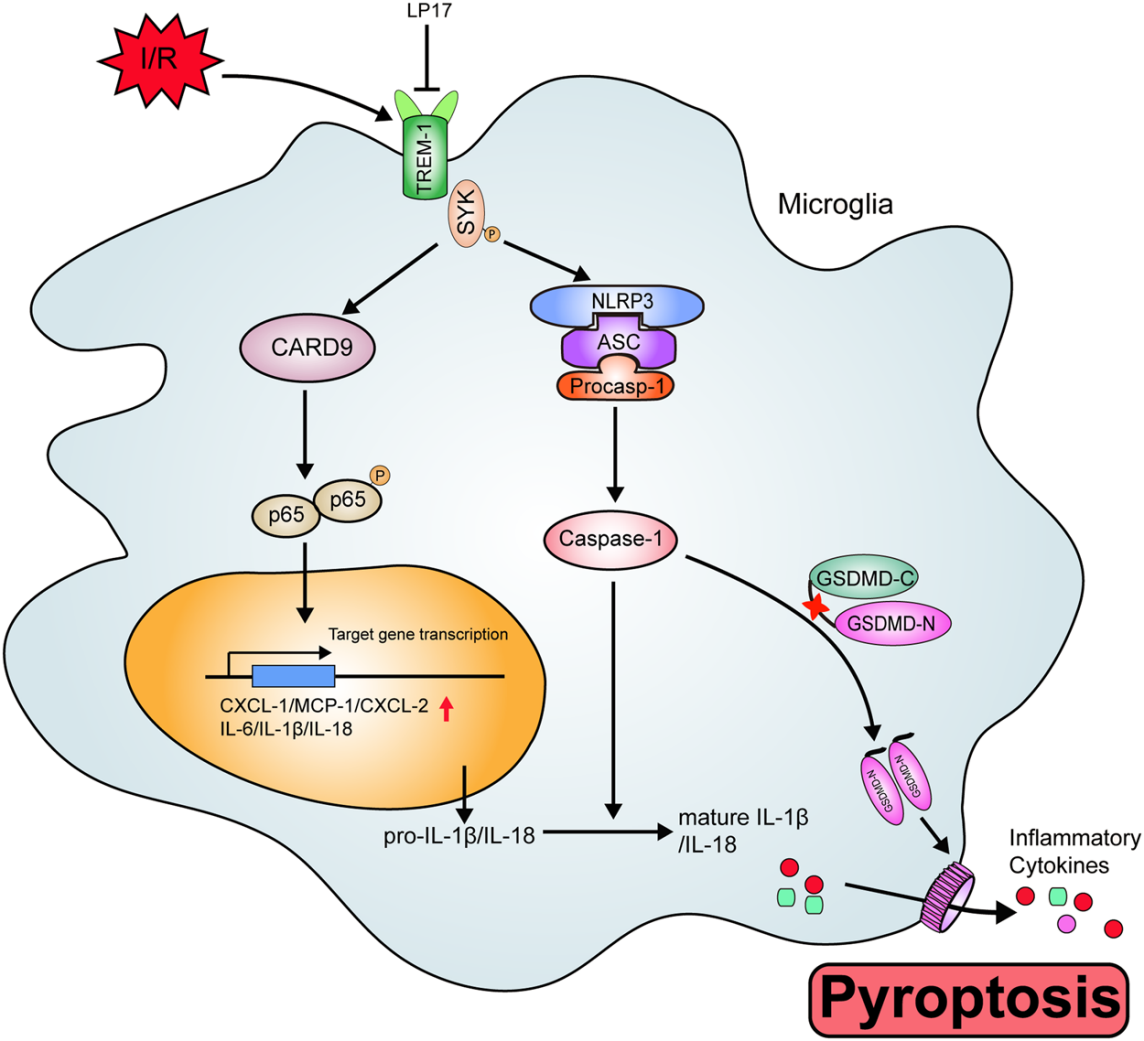
Schematic mechanism of microglial TREM-1 regulates post-ischemic neuroinflammation. TREM-1 level is upregulated following I/R injury. TREM-1-SYK association initiates SYK stimulation and mobilizes two downstream pathways, including CARD9/NF-κB and NLRP3/caspase-1, to generate inflammatory mediators and chemokines. In addition, the activated caspase-1 induced by SYK initiation cleaves GSDMD to release an N-terminal fragment, which forms pores on microglia, resulting in extracellular release of inflammatory factors such as IL-1β and IL-18. Pharmacological inhibition of TREM-1 alleviates inflammatory responses, resulting in ischemic stroke outcomes amelioration

Another major observation in this study was that TREM-1 can activate CARD9/NF-κB and NLRP3/caspase-1 pathways through interacting with SYK. We found TREM-1 was co-precipitated with microglial SYK both in vivo and in vitro. Microglial TREM-1 was co-localized with intracytoplasmic SYK. Mechanistically, as an immunoreceptor tyrosine-based activation motif-associated receptor, TREM-1 could couple with DNAX activation protein of 12 kDa (DAP12) through its charged lysine residue, subsequently recruiting and mobilizing SYK^42,43^. It has been reported that SYK initiation can active NF-κB signaling by controlling CARD9/Bcl-10 complex and activating NLRP3 inflammasome through SYK-dependent reactive oxygen species production^42^. CARD9/NF-κB is crucial for transcription of inflammatory genes and chemokines^10,44–46^, while NLRP3 is responsible for neutrophil infiltration^47^. Also, NLRP3 provides a molecular platform for procaspase-1 cleavage, which subsequently cleaves pro-IL-1β and pro-IL-18 into biologically mature IL-1β and mature IL-18, releasing into extracellular environment^48^. Our ELISA results confirmed that the extracellular levels of IL-1β and IL-18 were significantly increased 3 days after reperfusion. Recently, studies showed that GSDMD-executed pyroptosis is essential for IL-1β secretion^49,50^. GSDMD could release N-terminal fragment after cleaved by activated caspase-1, subsequently the N-terminal fragment binds to phosphoinositide in the plasma membrane and forms 12–14 nm membrane pore^32^. The formed pore serve as a gate for extracellular release of mature IL-1β^32^. Indeed, in this study, the expressions of GSDMD and GSDMD-N were drastically upregulated in microglia and more pores were detected on microglia membrane following ischemic stroke, indicating that pyroptosis occurs within microglia post-stroke. These data delineated an essential role of TREM-1 in orchestrating the inflammatory response following ischemic stroke (Fig. 10).

LP17 is an approved chemically synthesized TREM-1 inhibitory peptide comprising the complementary determining region-3 and the “F” β-strand of the extracellular domain of TREM-1^22^. Studies have shown that LP17 could compete with the nature ligand of TREM-1^22,51^. LP17 was administrated by intranasal delivery, a noninvasive method of drug delivery bypassing BBB to allow therapeutic substances direct access to the CNS^52^. The expression of TREM-1 and its association with SYK were substantially abrogated by LP17 or TREM-1 siRNA. So were NF-κB-induced inflammatory and chemotactic gene expressions and NLRP3-dependent pyroptosis. Blockade of TREM-1 not only suppressed microglial inflammation-associated injury both in vivo and in vitro, but also restored cell proliferation and synaptic plasticity in hippocampus. However, the possibility exists that microglial TREM-1 may be interacted with other proteins in ischemia-induced cerebral immune responses. Further studies are required to obtain more details in other possible associations mediated by TREM-1 in cerebral ischemic injury.

In conclusion, our data indicated that TREM-1 plays a critical role in post-ischemic neuroinflammation. We showed that microglial TREM-1 activated the CARD9/NF-κB signaling pathway and NLRP3 inflammasome via interaction with SYK, subsequently leading to inflammatory cytokines and chemokines production and pyroptosis. Blockade of TREM-1 can inhibit TREM-1/SYK pathway activation and subsequent inflammatory responses, rescuing stroke outcomes. Altogether, our data pointed to TREM-1 as a potential therapeutic target for treating ischemic stroke.

## Supplementary information

Supplementary Figure Legends

Supplementary Table S1

Supplementary Table S2

Supplementary Figure S1

Supplementary Figure S2

Supplementary Figure S3

Supplementary Figure S4

Supplementary Figure S5

Supplementary Figure S6

Supplementary Figure S7
