# Supplementary material for: Microglial TREM-1 receptor mediates neuroinflammatory injury via interaction with SYK in experimental ischemic stroke
Source: Cell Death Dis. 2019 Jul 19;10(8):555. doi: 10.1038/s41419-019-1777-9 (PMC6642102; doi:10.1038/s41419-019-1777-9)
Supplement: Supplementary file 1 — Supplementary Figure Legends [file 41419_2019_1777_MOESM1_ESM.doc]

**Supplementary Figure Legends**

**Supplementary Fig. S1** Changes of rCBF in the right MCA territory during and after MCAO.

During MCAO, monofilament insertion resulted in a sharp decrease of rCBF in the right MCA territory (≥ 75%), and returned to 90% of baseline after reperfusion. n = 6 in each group. Data are expressed as mean ± SEM.

**Supplementary Fig. S2** Immunostaining of TREM-1 with cell markers in ischemic penumbra 3 d after reperfusion.

TREM-1 was not co-localized with GFAP (astrocyte marker), NeuN (neuron marker), MBP (oligodendrocyte marker) or vWF (endothelial cell marker). Scale bar = 50 μm.

**Supplementary Fig. S3** Distribution of rhodamine labeled LP17 in mouse brain following intranasal delivery.

Mice were intranasally administrated with rhodamine labeled LP17 (1 mg/kg) for 3 days, and then brains were sectioned and labeled with DAPI. (**a**) Sagittal section of the brain. Captured regions are marked with dashed line boxes. (**b**) Rhodamine labeled LP17 signals were visualized in the olfactory bulb, cortex and hippocampus of mouse brain. Scale bar = 50 μm. LV: lateral ventricle; CC: corpus callosum; CTX: cortex.

**Supplementary Fig. S4** No significant differences were observed in (**a**) escape latency and (**b**) swimming speed during the cued trials. n = 10 in each group. Data are expressed as mean ± SEM.

**Supplementary Fig. S5** Statistical analysis for immunoblotting results of TREM-1, SYK and downstream components.

Quantitative histograms for the expressions of (**a**) TREM-1, p-SYK and SYK; (**b**) CARD9, p-p65 and p65; (**c**) NLRP3, ASC, procaspase-1, cleaved caspase-1, pro IL-1β, mature IL-1β and mature IL-18 in mice. Quantitative histograms for the expressions of (**d**) TREM-1, p-SYK and SYK; (**e**) CARD9, p-p65 and p65; (**f**) NLRP3, ASC, procaspase-1, cleaved caspase-1, pro IL-1β, mature IL-1β and mature IL-18 in microglia. (**g**) Quantitative histograms for the expressions of TREM1, SYK, p-SYK, CARD9, p-p65 and NLRP3 in siRNA transfected microglia. Data are expressed as mean ± SEM. n = 5 in each group. ****p* < 0.001 vs sham group; ^#^*p* < 0.05, ^##^*p* < 0.01, ^###^*p* < 0.001 vs MCAO group; ^†††^*p* < 0.001 vs control group; ^§^*p* < 0.05, ^§§^*p* < 0.01, ^§§§^*p* < 0.001 vs OGD/R group.

**Supplementary Fig. S6** Statistical analysis for immunoblotting results of TREM-1/SYK pathway and GSDMD.

(**a, b**) Quantitative histograms for the expressions of TREM1, SYK, CARD9, NLRP3 and cleaved caspase-1 in treated mice and cultured microglia. (**c, d**) Quantitative histograms for the expressions of GSDMD and GSDMD-N in treated mice and cultured microglia. Data are expressed as mean ± SEM. n = 5 in each group. ****p* < 0.001 vs sham group; ^#^*p* < 0.05, ^##^*p* < 0.01, ^###^*p* < 0.001 vs MCAO group; ^†††^*p*< 0.001 vs control group; ^§^*p* < 0.05, ^§§^*p* < 0.01, ^§§§^*p* < 0.001 vs OGD/R group.

**Supplementary Fig. S7** Quantitative analysis of GSDMD positive microglia after R406 treatment. Data are expressed as mean ± SEM. n = 5 in each group. ****p* < 0.001 vs sham group; ^###^*p* < 0.001 vs MCAO group.
