# Supplementary material for: Microglial TREM-1 receptor mediates neuroinflammatory injury via interaction with SYK in experimental ischemic stroke
Source: Cell Death Dis. 2019 Jul 19;10(8):555. doi: 10.1038/s41419-019-1777-9 (PMC6642102; doi:10.1038/s41419-019-1777-9)
Supplement: Supplementary file 2 — Supplementary Table S1 [file 41419_2019_1777_MOESM2_ESM.doc]

**Supplementary Table S1** Primers used in real-time PCR

| Gene | Sense Primer (5'-3') | Antisense Primer (3'-5') |
| --- | --- | --- |
| TREM-1 | ACCGCAGTGGGCTTGGGTAGGG | GAGGAAGGCTGGGCTCTGGGGACT |
| NLRP3 | GCATTGCTTCGTAGATAGAGG | GATGAAGGACCCACAGTGTAA |
| IL-1β | TTGTTCATCTCGGAGCCTGTA | AGCACCTTCTTTTCCTTCATC |
| IL-6 | GCACTAGGTTTGCCGAGTAGA | AAGCTGGAGTCACAGAAGGAG |
| NFKB2 | GGGCAGACCAGTGTCATTGAG | CCATGCCGATCCAGCAGAG |
| iNOS | AATGCCCGTACCAGGCCCAAT | GGTCACCTACCGCACCCGAGAT |
| CD16 | ACTGTGGTTGGCTTTTGGGAT | GAGTGATTTCTGACTGGCTGCTG |
| CD32 | AGGGCTGTCTGTACTCACCTACTTC | CTGTCACTGGGATTGCTGTCG |
| Arg-1 | TCTTTGGCAGATATGCAGGGA | CACAGTCTGGCAGTTGGAAGC |
| CD206 | AAAGCCACTTCCCTTCAACAT | ACTGCGTGGATTCCTTTCTAT |
| IL-10 | GCCTGGGGCATCACTTCTACC | CTGGACAACATACTGCTAACCGAC |
| IL-18 | ACCACTTTGGCAGACTTCACT | ACACAGGCGGGTTTCTTTTG |
| CD11b | GTATAGGCCAGCAGTGATGAG | GCAGGAGTCGTATGTGAGGTC |
| CXCL-2 | GGCTTCAGGGTCAAGGCAAAC | ACCAACCACCAGGCTACAGGG |
| CCL-7 | CAAGGCTTTGGAGTTGGGGTT | AGAAGGGCATGGAAGTCTGCG |
| MCP-1 | TTGAGGTGGTTGTGGAAAAGG | GTGCTGACCCCAAGAAGGAAT |
| CXCL-1 | GTGGCTATGACTTCGGTTTGG | ACCAGTTCCAGCACTCCAGAC |
| GAPDH | AAGAAGGTGGTGAAGCAGG | GAAGGTGGAAGAGTGGGAGT |
