# Supplementary material for: Microglial TREM-1 receptor mediates neuroinflammatory injury via interaction with SYK in experimental ischemic stroke
Source: Cell Death Dis. 2019 Jul 19;10(8):555. doi: 10.1038/s41419-019-1777-9 (PMC6642102; doi:10.1038/s41419-019-1777-9)
Supplement: Supplementary file 3 — Supplementary Table S2 [file 41419_2019_1777_MOESM3_ESM.pdf]

Supplementary Table S2 The differential expressed immune genes in the peri-infarct region at 24 h post-modeling

gene_id	GeneSymbol	length	Sham1.rawreads	Sham1.FPKM	Sham2.rawreads	Sham2.FPKM	Sham3.rawreads	Sham3.FPKM	Sham4.rawreads	Sham4.FPKM	Sham5.rawread	Sham5.FPKM	MCAO1d1.rawreads	MCAO1d1.FPKM	MCAO1d2.rawread	MCAO1d2.FPKM	MCAO1d3.rawreads	MCAO1d3.FPKM	MCAO1d4.rawreads	MCAO1d4.FPKM	MCAO1d5.rawread	MCAO1d5.FPKM	logFC	logCPM	p-value	FDR	Regulation
ENSMUSG00000029380	Cxcl1	1875	0	0	0	0	0	0	2	0.04	0	0	168	2.85	102	1.77	194	3.48	178	3.1	109	2.48	8.1095666	1.946893455	1.98E-58	9.11E-54	Ups
ENSMUSG00000035385	Ccl2	1883	5	0.12	0	0	1	0.03	0	0	1	0.03	188	4.72	248	6.38	308	8.16	226	5.83	68	2.29	7.0094804	2.382518137	7.74E-41	1.78E-36	Ups
ENSMUSG00000000982	Ccl3	1512	12	0.74	4	0.25	4	0.25	1	0.07	5	0.37	147	9.19	253	16.23	212	14.01	129	8.29	79	6.62	4.8816658	2.101594865	6.45E-30	4.95E-26	Ups
ENSMUSG00000018930	Ccl4	2100	6	0.43	1	0.07	3	0.22	2	0.16	8	0.69	153	11.1	287	21.37	233	17.87	119	8.88	73	7.1	5.3022097	2.161953459	1.31E-28	7.54E-25	Ups
ENSMUSG00000058427	Cxcl2	2068	0	0	0	0	0	0	0	0	0	0	28	0.26	38	0.36	92	0.9	35	0.33	41	0.51	8.5397927	0.360542583	4.43E-24	1.57E-20	Ups
ENSMUSG00000005413	Hmox1	7006	119	2.03	130	2.21	54	0.95	67	1.26	87	1.8	1499	25.93	4054	71.93	2507	45.82	1177	20.93	426	9.88	4.3098493	5.627982812	1.30E-23	3.99E-20	Ups
ENSMUSG00000051439	Cd14	1663	80	2.41	34	1.02	54	1.67	38	1.25	37	1.34	472	14.38	1678	52.45	1032	33.22	474	14.85	344	14.06	3.9807988	4.400180495	1.84E-23	5.29E-20	Ups
ENSMUSG00000027832	Ptx3	5896	6	0.09	0	0	5	0.08	2	0.03	5	0.09	251	3.9	483	7.69	572	9.39	287	4.58	33	0.69	6.3372409	3.008338956	2.28E-22	6.18E-19	Ups
ENSMUSG00000035673	Sbno2	45283	582	6.51	540	6.01	642	7.37	543	6.67	465	6.29	1559	17.66	1668	19.39	1759	21.06	1504	17.52	953	14.48	1.3665535	5.681118246	4.61E-22	1.12E-18	Ups
ENSMUSG00000027995	Tlr2	5496	15	0.24	23	0.36	14	0.23	15	0.26	11	0.21	129	2.05	270	4.4	184	3.09	76	1.24	103	2.19	3.2333447	2.110361055	4.75E-18	8.76E-15	Ups
ENSMUSG00000050335	Lgals3	18410	10	0.17	5	0.08	14	0.24	6	0.11	8	0.16	141	2.42	455	8.02	321	5.83	86	1.52	56	1.29	4.5235984	2.464318219	8.28E-17	1.27E-13	Ups
ENSMUSG00000025746	Il6	6868	5	0.06	0	0	0	0	0	0	1	0.01	74	0.87	175	2.1	169	2.09	49	0.59	20	0.31	6.1185962	1.333827947	1.09E-16	1.56E-13	Ups
ENSMUSG00000062593	Lilrb4	15974	30	0.74	6	0.15	11	0.28	10	0.27	5	0.15	314	7.82	1067	27.28	549	14.45	201	5.15	75	2.51	5.0719252	3.48038498	2.26E-16	3.06E-13	Ups
ENSMUSG00000053175	Bcl3	14309	12	0.16	6	0.08	18	0.24	10	0.14	8	0.13	145	1.91	193	2.61	158	2.2	91	1.23	35	0.62	3.4268078	1.774213729	2.58E-16	3.39E-13	Ups
ENSMUSG00000020676	Ccl11	5133	10	0.44	1	0.04	0	0	0	0	1	0.05	115	5.09	166	7.54	282	13.19	81	3.69	31	1.84	5.6656507	1.789166854	3.05E-15	3.51E-12	Ups
ENSMUSG00000038418	Egr1	5162	4594	28.9	9102	56.98	7797	50.32	8115	56	5524	41.97	2565	16.34	2474	16.16	3330	22.41	3436	22.5	1617	13.81	-1.45237	7.954212535	3.80E-15	4.27E-12	Down
ENSMUSG00000047798	Cd300lf	17779	14	0.1	9	0.06	2	0.01	8	0.06	3	0.03	39	0.27	172	1.24	82	0.61	137	0.99	111	1.05	3.8844979	1.609866857	6.92E-15	7.24E-12	Ups
ENSMUSG00000034855	Cxcl10	2252	0	0	0	0	3	0.03	0	0	4	0.05	42	0.42	178	1.84	56	0.59	88	0.91	21	0.28	5.5639229	1.031905648	9.05E-15	9.27E-12	Ups
ENSMUSG00000034394	Lif	14958	18	0.19	15	0.16	15	0.16	20	0.23	13	0.17	132	1.42	153	1.69	101	1.15	241	2.67	40	0.58	2.9422647	1.919011264	1.76E-14	1.76E-11	Ups
ENSMUSG00000024521	Pmaip1	6947	59	1.05	56	1	45	0.83	40	0.78	44	0.95	328	5.94	213	3.96	270	5.16	121	2.25	309	7.5	2.3327544	2.948699265	3.24E-14	3.04E-11	Ups
ENSMUSG00000035373	Ccl7	1814	3	0.18	0	0	2	0.12	0	0	2	0.14	71	4.25	132	8.1	82	5.18	86	5.29	5	0.4	5.5206248	0.979935391	3.82E-14	3.52E-11	Ups
ENSMUSG00000059187	Fam19a1	544045	982	7.14	1000	7.24	1208	9.02	1035	8.26	878	7.72	513	3.78	344	2.6	375	2.92	607	4.6	336	3.32	-1.281768	5.221497903	5.25E-14	4.65E-11	Down
ENSMUSG00000024401	Tnf	2627	2	0.03	0	0	0	0	0	0	0	0	17	0.27	54	0.87	40	0.66	15	0.24	8	0.17	5.6168519	-0.358109273	3.19E-12	2.19E-09	Ups
ENSMUSG00000046879	Irgm1	9716	182	1.18	190	1.22	157	1.04	122	0.87	201	1.57	428	2.81	1124	7.56	511	3.54	598	4.03	555	4.88	1.875789	4.380446672	3.58E-12	2.40E-09	Ups
ENSMUSG00000061353	Cxcl12	12833	751	2.74	812	2.95	902	3.38	790	3.17	879	3.88	328	1.21	310	1.18	240	0.94	454	1.73	335	1.66	-1.347016	4.909373692	3.59E-12	2.40E-09	Down
ENSMUSG00000029304	Spp1	5933	230	2.03	101	0.89	307	2.77	212	2.05	153	1.63	1658	14.78	8342	76.3	2584	24.34	1362	12.48	343	4.1	3.749504	6.223676863	7.52E-12	4.65E-09	Ups
ENSMUSG00000039232	Stx11	24277	4	0.08	0	0	3	0.06	3	0.06	6	0.14	26	0.52	23	0.47	35	0.74	49	1.01	24	0.64	3.184766	-0.036696633	7.75E-12	4.70E-09	Ups
ENSMUSG00000021453	Gadd45g	1791	179	4.38	226	5.5	356	8.93	318	8.53	158	4.67	660	16.34	1123	28.53	843	22.06	679	17.29	552	18.33	1.5989386	4.686701529	1.36E-11	7.83E-09	Ups
ENSMUSG00000038067	Csf3	2367	3	0.1	1	0.03	0	0	2	0.07	3	0.12	40	1.36	6	0.21	84	3.01	85	2.96	125	5.68	5.1732801	0.951032741	1.63E-11	9.28E-09	Ups
ENSMUSG00000019122	Ccl9	5718	24	0.35	47	0.69	41	0.62	28	0.45	27	0.48	264	3.92	993	15.14	439	6.89	220	3.36	57	1.14	3.4708938	3.401593955	1.79E-11	1.00E-08	Ups
ENSMUSG00000049130	C5ar1	12798	47	0.44	13	0.12	7	0.07	7	0.07	10	0.11	194	1.85	166	1.62	210	2.12	153	1.5	58	0.74	3.138451	2.120680517	2.69E-11	1.46E-08	Ups
ENSMUSG00000035352	Ccl12	1556	17	0.89	3	0.16	7	0.38	9	0.52	5	0.32	207	11.02	941	51.37	289	16.25	130	7.11	12	0.86	5.1597657	2.996201459	2.69E-11	1.46E-08	Ups
ENSMUSG00000028874	Fgr	27816	3	0.03	3	0.03	1	0.01	1	0.01	0	0	16	0.16	66	0.69	40	0.43	23	0.24	13	0.18	4.1506307	-0.100681571	4.19E-11	2.12E-08	Ups
ENSMUSG00000025163	Cd7	2672	47	1.54	31	1.01	34	1.14	29	1.04	22	0.87	92	3.04	141	4.78	101	3.53	118	4.01	91	4.04	1.7009548	1.87719097	5.12E-11	2.56E-08	Ups
ENSMUSG00000027611	Procr	4355	11	0.14	23	0.29	13	0.17	16	0.22	16	0.24	71	0.9	120	1.56	106	1.42	82	1.07	31	0.53	2.2842174	1.341522303	1.14E-10	5.19E-08	Ups
ENSMUSG00000061132	Blnk	65609	122	0.92	144	1.09	105	0.82	133	1.1	91	0.83	289	2.22	404	3.18	388	3.14	302	2.38	532	5.47	1.6974157	3.715379915	1.82E-10	7.84E-08	Ups
ENSMUSG00000016524	Il19	7460	2	0.03	0	0	0	0	0	0	0	0	6	0.1	16	0.27	14	0.24	18	0.3	10	0.22	4.5898737	-1.198785289	5.39E-10	2.20E-07	Ups
ENSMUSG00000048572	Tmem252	5654	117	2.25	29	0.55	43	0.85	45	0.95	30	0.7	183	3.56	274	5.47	316	6.5	197	3.94	357	9.31	2.3518559	3.058437667	9.45E-10	3.75E-07	Ups
ENSMUSG00000073418	C4b	15503	442	3.32	199	1.49	351	2.71	166	1.37	185	1.68	776	5.9	1308	10.21	1243	9.99	863	6.75	414	4.22	1.7208022	4.88708135	9.89E-10	3.87E-07	Ups
ENSMUSG00000085609	1700016P0	5074	162	8.88	285	15.55	368	20.69	302	18.16	158	10.46	83	4.61	27	1.54	35	2.05	103	5.88	45	3.35	-2.167419	3.018500715	1.02E-09	3.94E-07	Down
ENSMUSG00000032508	Myd88	5478	122	1.25	88	0.9	135	1.42	118	1.33	79	0.98	324	3.36	419	4.45	350	3.83	262	2.79	163	2.27	1.4230372	3.376686754	1.36E-09	5.09E-07	Ups
ENSMUSG00000061531	Tmem236	47471	5	0.06	3	0.04	6	0.08	3	0.04	1	0.02	33	0.43	16	0.21	41	0.57	18	0.24	27	0.47	2.8378991	-0.191880404	1.41E-09	5.25E-07	Ups
ENSMUSG00000048163	Selplg	14109	436	3.6	440	3.62	494	4.18	422	3.82	351	3.5	241	2.01	257	2.2	156	1.38	193	1.66	98	1.1	-1.24811	3.982019918	1.65E-09	6.04E-07	Down
ENSMUSG00000021070	Bdkrb2	29900	7	0.2	3	0.08	2	0.06	4	0.12	3	0.1	15	0.43	34	0.99	28	0.84	28	0.82	20	0.76	2.6394608	-0.267383409	1.94E-09	6.99E-07	Ups
ENSMUSG00000032691	Nlrp3	25389	23	0.21	23	0.21	21	0.2	20	0.2	14	0.16	56	0.52	83	0.79	75	0.74	59	0.56	44	0.55	1.5978084	1.142415453	6.15E-09	1.95E-06	Ups
ENSMUSG00000030144	Clec4d	13162	2	0.07	0	0	0	0	0	0	0	0	7	0.25	81	2.98	25	0.95	13	0.48	8	0.38	5.6316248	-0.351944663	6.68E-09	2.11E-06	Ups
ENSMUSG00000052125	F730043M1	35887	542	4.15	560	4.27	659	5.18	536	4.5	554	5.13	337	2.61	239	1.9	339	2.78	389	3.1	188	1.96	-1.001277	4.475624589	1.57E-08	4.67E-06	Down
ENSMUSG00000046818	Ddit4l	6722	1470	21.28	1321	19.04	781	11.6	849	13.48	923	16.14	566	8.3	533	8.02	508	7.87	566	8.53	195	3.83	-1.254171	5.293469811	1.71E-08	4.96E-06	Down
ENSMUSG00000050505	Pcdh20	6654	652	3.58	471	2.58	753	4.25	736	4.44	763	5.07	426	2.37	350	2	314	1.85	284	1.63	140	1.05	-1.249601	4.649820171	1.77E-08	5.08E-06	Down
ENSMUSG00000078137	Ankrd63	4861	502	4.88	630	6.1	579	5.78	483	5.16	434	5.1	162	1.6	124	1.25	201	2.09	335	3.39	208	2.75	-1.375938	4.238676752	1.91E-08	5.32E-06	Down
ENSMUSG00000030748	Il4ra	27191	244	2.27	227	2.1	170	1.62	126	1.28	160	1.8	433	4.07	467	4.51	550	5.47	430	4.16	272	3.43	1.1587364	3.958592745	2.61E-08	6.96E-06	Ups
ENSMUSG00000022537	Tmem44	38988	2195	7.52	1498	5.11	2854	10.03	2567	9.64	2694	11.15	1353	4.69	656	2.33	1100	4.03	1327	4.73	1034	4.81	-1.162538	6.479734794	2.82E-08	7.37E-06	Down
ENSMUSG00000058755	Osm	4607	0	0	7	0.13	6	0.11	2	0.04	0	0	21	0.39	39	0.75	54	1.07	20	0.39	16	0.4	3.2256983	-0.107621405	2.89E-08	7.53E-06	Ups
ENSMUSG00000025225	Nfkb2	7354	47	0.66	54	0.75	46	0.66	44	0.67	43	0.73	119	1.68	181	2.63	145	2.17	110	1.6	74	1.4	1.3630483	2.149698547	4.03E-08	9.99E-06	Ups
ENSMUSG00000046733	Gprc5a	19059	6	0.14	1	0.02	4	0.09	5	0.12	1	0.03	20	0.46	9	0.21	36	0.87	85	2.01	50	1.54	3.513883	0.276680968	4.25E-08	1.04E-05	Ups
ENSMUSG00000006014	Prg4	16754	461	3.01	163	1.06	286	1.92	178	1.28	117	0.92	914	6.05	448	3.04	1003	7.01	4022	27.36	700	6.21	2.5060573	5.357834168	8.55E-08	1.98E-05	Ups
ENSMUSG00000079293	Clec7a	11187	1	0.02	3	0.05	3	0.05	1	0.02	1	0.02	18	0.3	94	1.62	44	0.78	23	0.4	2	0.05	4.1557185	0.060625378	8.61E-08	1.98E-05	Ups
ENSMUSG00000020286	1700093K2	4953	15	0.29	20	0.38	9	0.18	11	0.23	13	0.3	35	0.68	46	0.91	51	1.04	60	1.19	80	2.08	1.9979614	0.896255627	1.05E-07	2.39E-05	Ups
ENSMUSG00000035394	Cfap53	76897	1	0.01	0	0	1	0.01	3	0.04	2	0.03	19	0.24	6	0.08	18	0.24	16	0.21	51	0.85	3.9162599	-0.483103666	1.13E-07	2.54E-05	Ups
ENSMUSG00000033777	Tlr13	17290	54	0.61	36	0.41	48	0.56	29	0.36	39	0.53	105	1.2	315	3.7	187	2.26	102	1.2	83	1.28	1.8830896	2.351880538	1.16E-07	2.57E-05	Ups
ENSMUSG00000072591	5930412G1	21588	98	0.93	105	0.99	69	0.67	109	1.13	110	1.26	47	0.45	51	0.5	40	0.41	36	0.36	19	0.24	-1.42455	1.851546055	1.41E-07	3.05E-05	Down
ENSMUSG00000037405	Icam1	12838	63	1.17	77	1.43	93	1.78	72	1.47	40	0.9	172	3.24	243	4.7	223	4.44	210	4.07	90	2.28	1.3821681	2.699140021	1.42E-07	3.06E-05	Ups
ENSMUSG00000060962	Dmkn	17308	16	0.19	3	0.04	12	0.15	16	0.21	21	0.3	58	0.7	59	0.73	67	0.85	155	1.92	38	0.61	2.3655755	1.218476557	1.56E-07	3.30E-05	Ups
ENSMUSG00000059108	Ifitm6	1081	2	0.17	3	0.26	3	0.27	2	0.19	1	0.11	15	1.33	16	1.45	18	1.68	16	1.46	7	0.83	2.5851835	-0.947651702	1.76E-07	3.71E-05	Ups
ENSMUSG00000029379	Cxcl3	3498	1	0.01	0	0	0	0	0	0	0	0	3	0.03	25	0.27	15	0.17	12	0.13	3	0.04	5.1164605	-1.328818874	1.86E-07	3.86E-05	Ups
ENSMUSG00000033107	Rnf125	39224	22	0.78	5	0.18	5	0.18	6	0.23	4	0.17	33	1.18	30	1.1	76	2.88	42	1.55	55	2.65	2.47391	0.603689919	2.23E-07	4.56E-05	Ups
ENSMUSG00000032921	Odf4	5247	2	0.03	8	0.14	6	0.11	4	0.08	4	0.08	18	0.32	22	0.4	26	0.48	21	0.38	20	0.47	2.0913327	-0.383627191	2.26E-07	4.61E-05	Ups
ENSMUSG00000040552	C3ar1	9024	82	0.9	51	0.56	37	0.42	54	0.65	27	0.36	206	2.29	614	7.01	249	2.93	143	1.64	73	1.09	2.2869509	2.943097081	2.35E-07	4.76E-05	Ups
ENSMUSG00000025491	Ifitm1	2605	77	2.79	28	1.01	71	2.64	39	1.55	22	0.96	168	6.17	424	15.96	176	6.82	182	6.87	81	3.99	2.0693811	2.675988891	2.66E-07	5.30E-05	Ups
ENSMUSG00000025591	Tma16	12201	245	2.03	158	1.3	245	2.09	221	2.01	188	1.88	453	3.8	379	3.27	505	4.48	429	3.7	607	6.84	1.1615079	4.153755556	3.86E-07	7.32E-05	Ups
ENSMUSG00000059089	Fcgr4	10842	12	0.45	1	0.04	1	0.04	2	0.08	5	0.23	29	1.11	83	3.25	34	1.37	43	1.69	10	0.51	3.1233254	0.258967307	4.26E-07	8.02E-05	Ups
ENSMUSG00000078945	Naip2	58030	21	0.18	12	0.1	10	0.09	9	0.09	8	0.08	41	0.36	86	0.78	68	0.64	33	0.3	24	0.29	2.0031012	0.73373783	4.90E-07	8.93E-05	Ups
ENSMUSG00000028362	Tnfsf8	30040	8	0.1	1	0.01	2	0.03	0	0	0	0	44	0.55	28	0.36	15	0.2	11	0.14	17	0.29	3.27931	-0.447369136	4.96E-07	8.96E-05	Ups
ENSMUSG00000051435	Fhad1	124645	93	0.59	92	0.58	94	0.61	86	0.6	86	0.66	166	1.07	286	1.88	198	1.34	175	1.16	200	1.72	1.1546553	2.934500166	5.17E-07	9.31E-05	Ups
ENSMUSG00000039236	Isg20	6939	6	0.09	11	0.17	7	0.11	10	0.17	8	0.15	35	0.54	58	0.92	30	0.49	38	0.6	14	0.29	1.967538	0.248843274	5.93E-07	0.000105	Ups
ENSMUSG00000044583	Tlr7	25630	95	0.39	57	0.23	75	0.32	34	0.15	39	0.19	131	0.55	240	1.02	162	0.71	144	0.62	211	1.18	1.5749295	2.632184242	5.94E-07	0.000105	Ups
ENSMUSG00000024679	Ms4a6d	18246	54	1.1	10	0.2	32	0.67	21	0.47	13	0.32	130	2.68	613	12.97	167	3.64	125	2.65	33	0.91	2.9693119	2.582781794	6.36E-07	0.000112	Ups
ENSMUSG00000027514	Zbp1	12312	3	0.03	4	0.04	1	0.01	3	0.03	3	0.04	18	0.18	184	1.87	25	0.26	14	0.14	9	0.12	4.0197582	0.50470657	6.41E-07	0.000113	Ups
ENSMUSG00000054901	Arhgef33	81282	35	0.51	41	0.59	33	0.49	33	0.53	22	0.39	71	1.04	68	1.02	83	1.29	102	1.54	75	1.48	1.2493667	1.565566126	9.60E-07	0.000161	Ups
ENSMUSG00000027718	Il21	9878	1	0.01	2	0.02	4	0.03	2	0.02	2	0.02	10	0.08	15	0.13	18	0.16	17	0.15	8	0.09	2.5075581	-0.999928755	9.71E-07	0.000162	Ups
ENSMUSG00000047730	Fcgbp	49627	5	0.02	7	0.02	6	0.02	6	0.02	1	0	24	0.08	23	0.08	41	0.15	25	0.09	9	0.04	2.1932294	-0.259927718	1.18E-06	0.000192	Ups
ENSMUSG00000074151	Nlrc5	54510	7	0.03	20	0.08	26	0.1	15	0.06	13	0.06	49	0.19	146	0.58	68	0.28	73	0.29	25	0.13	2.0775296	1.204891335	1.71E-06	0.000261	Ups
ENSMUSG00000048636	A730049H0	30697	0	0	1	0.01	2	0.02	4	0.05	1	0.01	15	0.17	27	0.32	35	0.43	8	0.1	4	0.06	3.2885112	-0.770526039	1.72E-06	0.000261	Ups
ENSMUSG00000030789	Itgax	21110	3	0.04	4	0.05	6	0.07	2	0.03	4	0.06	26	0.31	78	0.95	51	0.64	12	0.15	5	0.08	3.0478772	0.071000471	1.73E-06	0.000262	Ups
ENSMUSG00000015340	Cybb	52520	12	0.09	14	0.1	8	0.06	16	0.13	5	0.05	42	0.32	342	2.67	104	0.84	31	0.24	16	0.16	3.1962887	1.595241056	1.88E-06	0.000284	Ups
ENSMUSG00000030142	Clec4e	8082	0	0	0	0	0	0	1	0	0	0	5	0.02	61	0.23	17	0.07	2	0.01	3	0.01	5.7022273	-0.867155351	2.21E-06	0.000322	Ups
ENSMUSG00000075510	Fam187a	1563	7	0.21	2	0.06	2	0.06	3	0.1	2	0.07	14	0.43	9	0.28	25	0.81	22	0.69	16	0.66	2.3467711	-0.693550132	2.23E-06	0.000323	Ups
ENSMUSG00000000202	Btbd17	4729	563	2.78	558	2.74	526	2.66	474	2.57	526	3.14	339	1.69	414	2.12	219	1.16	187	0.96	126	0.84	-1.119382	4.331772391	2.70E-06	0.000377	Down
ENSMUSG00000038059	Smim3	27791	348	8.3	283	6.72	158	3.86	181	4.73	116	3.34	415	10.02	690	17.09	478	12.19	500	12.41	473	15.31	1.2230812	4.216821854	3.19E-06	0.000435	Ups
ENSMUSG00000004698	Hdac9	157387	653	6.98	650	6.92	823	9.03	782	9.18	630	8.14	401	4.34	469	5.21	310	3.55	492	5.48	140	2.03	-1.049777	4.768789613	3.22E-06	0.000438	Down
ENSMUSG00000034641	Cd300ld	7649	2	0.04	0	0	0	0	1	0.02	1	0.02	12	0.22	53	0.98	31	0.59	1	0.02	6	0.15	4.4099879	-0.648556328	3.32E-06	0.000448	Ups
ENSMUSG00000090622	A930033H1	3337	81	1.29	77	1.22	80	1.31	78	1.36	81	1.56	47	0.76	22	0.36	41	0.7	47	0.78	15	0.32	-1.285344	1.585036482	3.50E-06	0.000467	Down
ENSMUSG00000089837	Npcd	44563	32	1.04	6	0.19	9	0.3	10	0.36	6	0.24	37	1.22	34	1.15	55	1.92	86	2.91	164	7.25	2.6618684	1.284132918	4.41E-06	0.000565	Ups
ENSMUSG00000010830	Kdelr3	11332	8	0.27	7	0.23	12	0.41	8	0.29	9	0.36	19	0.64	40	1.39	27	0.97	32	1.11	28	1.27	1.6837729	0.096953623	4.44E-06	0.000566	Ups
ENSMUSG00000051832	E230016K2	41638	3	0.04	1	0.01	2	0.03	1	0.01	0	0	9	0.12	6	0.08	11	0.16	12	0.17	31	0.57	3.2648215	-1.009672145	5.51E-06	0.000677	Ups
ENSMUSG00000035678	Tnfsf9	2373	2	0.08	8	0.31	7	0.28	14	0.59	2	0.09	43	1.67	33	1.32	50	2.06	15	0.6	19	0.99	2.192996	0.09601911	6.28E-06	0.000756	Ups
ENSMUSG00000085156	Gm11974	3170	30	0.65	53	1.14	48	1.06	34	0.8	33	0.86	124	2.7	103	2.3	117	2.69	74	1.66	56	1.64	1.1931582	1.794398548	6.70E-06	0.000798	Ups
ENSMUSG00000020395	Itk	64366	9	0.07	5	0.04	11	0.09	7	0.06	6	0.06	26	0.2	74	0.59	22	0.18	22	0.18	21	0.22	2.0572431	0.172015621	6.77E-06	0.000802	Ups
ENSMUSG00000025492	Ifitm3	1185	197	13.74	100	6.94	173	12.38	138	10.56	102	8.6	314	22.18	1454	105.37	439	32.77	351	25.49	172	16.29	1.8795213	4.103485005	7.38E-06	0.000859	Ups
ENSMUSG00000040441	Slc26a10	8220	73	1.56	98	2.08	71	1.56	54	1.27	54	1.39	160	3.46	117	2.6	174	3.98	142	3.16	150	4.35	1.0592087	2.501403996	7.98E-06	0.000912	Ups
ENSMUSG00000062007	Hsh2d	11328	0	0	0	0	1	0.02	0	0	0	0	13	0.3	3	0.07	8	0.19	3	0.07	5	0.15	4.2804372	-1.899880677	8.17E-06	0.00093	Ups
ENSMUSG00000021903	Galnt15	33209	110	0.58	18	0.09	63	0.34	48	0.28	39	0.25	150	0.8	138	0.75	172	0.96	194	1.06	248	1.76	1.7141326	2.631192763	8.30E-06	0.00094	Ups
ENSMUSG00000024235	Map3k8	21689	59	0.37	46	0.28	40	0.25	43	0.29	52	0.39	87	0.55	112	0.72	117	0.78	104	0.67	96	0.81	1.0620729	1.984278948	8.69E-06	0.000977	Ups
ENSMUSG00000033576	Apol6	12379	3	0.02	1	0.01	0	0	1	0.01	5	0.04	12	0.07	10	0.06	13	0.08	12	0.07	25	0.2	2.769821	-0.92929321	1.10E-05	0.001172	Ups
ENSMUSG00000066682	Pilrb2	5989	0	0	1	0.01	0	0	1	0.02	0	0	3	0.04	19	0.29	11	0.17	3	0.05	6	0.12	3.9897638	-1.622448148	1.10E-05	0.001175	Ups
ENSMUSG00000026822	Lcn2	3620	326	3.17	16	0.15	137	1.37	140	1.49	1	0.01	673	6.62	1506	15.2	1212	12.6	731	7.39	1361	17.96	3.1919374	4.981633462	1.14E-05	0.001201	Ups
ENSMUSG00000024778	Fas	37112	57	1.5	30	0.78	39	1.05	31	0.89	13	0.41	69	1.83	94	2.56	99	2.78	90	2.46	90	3.21	1.3707184	1.683509089	1.18E-05	0.001233	Ups
ENSMUSG00000040663	Clcf1	9253	37	0.28	35	0.27	53	0.42	41	0.34	37	0.34	94	0.73	381	3.03	144	1.18	120	0.96	44	0.46	1.8665105	2.323615463	1.21E-05	0.001252	Ups
ENSMUSG00000032776	Mctp2	228766	33	0.27	30	0.24	39	0.33	37	0.33	32	0.32	85	0.7	93	0.79	72	0.63	62	0.53	132	1.46	1.3808792	1.719248945	1.24E-05	0.001278	Ups
ENSMUSG00000019832	Rab32	13223	41	0.92	22	0.49	33	0.76	35	0.86	18	0.49	65	1.48	134	3.12	84	2.02	63	1.47	53	1.61	1.3755964	1.517781938	1.43E-05	0.00142	Ups
ENSMUSG00000091017	Fam71a	2234	0	0	0	0	0	0	0	0	0	0	2	0.04	13	0.29	17	0.39	3	0.07	1	0.03	5.8164065	-1.823599559	1.43E-05	0.001423	Ups
ENSMUSG00000027524	Edn3	23424	104	0.78	54	0.4	85	0.65	63	0.52	90	0.81	44	0.33	37	0.29	38	0.3	38	0.29	29	0.29	-1.144348	1.62471285	1.51E-05	0.001478	Down
ENSMUSG00000040528	Milr1	17569	6	0.06	1	0.01	8	0.08	2	0.02	2	0.02	29	0.27	106	1.02	26	0.26	11	0.11	6	0.08	3.1218508	0.112966094	1.62E-05	0.001574	Ups
ENSMUSG00000045667	Smtnl2	22550	18	0.15	12	0.1	14	0.12	5	0.04	11	0.11	38	0.31	14	0.12	43	0.37	57	0.48	323	3.55	3.1572279	1.635041585	1.68E-05	0.001627	Ups
ENSMUSG00000027399	Il1a	10363	19	0.46	6	0.14	27	0.66	19	0.5	14	0.41	66	1.6	35	0.87	57	1.46	42	1.05	45	1.46	1.4792103	0.825034153	1.92E-05	0.00182	Ups
ENSMUSG00000058470	Gm8369	26640	3	0.02	1	0.01	2	0.01	2	0.01	6	0.05	18	0.12	18	0.12	11	0.08	10	0.07	12	0.11	2.1940188	-0.934909789	2.11E-05	0.001976	Ups
ENSMUSG00000033213	AA467197	4206	6	0.15	2	0.05	5	0.12	7	0.19	4	0.12	20	0.49	86	2.17	16	0.42	20	0.51	9	0.3	2.5504209	-0.030728335	2.25E-05	0.002081	Ups
ENSMUSG00000018927	Ccl6	5206	20	0.37	8	0.15	26	0.5	17	0.35	26	0.58	113	2.13	237	4.57	115	2.29	51	0.99	12	0.3	2.3209008	1.668562299	2.25E-05	0.002081	Ups
ENSMUSG00000038917	3930402G2	4031	6	0.1	3	0.05	3	0.05	10	0.18	5	0.1	13	0.22	20	0.35	28	0.5	22	0.38	18	0.41	1.8350026	-0.410753564	2.57E-05	0.002342	Ups
ENSMUSG00000032564	Cpne4	487259	951	7.65	1116	8.93	1420	11.72	1292	11.4	932	9.05	443	3.61	330	2.76	536	4.61	994	8.32	564	6.16	-1.02279	5.463679743	2.57E-05	0.002344	Down
ENSMUSG00000071656	Lrrn4cl	3125	23	0.42	28	0.51	31	0.58	27	0.54	15	0.33	64	1.19	51	0.97	59	1.16	65	1.24	38	0.94	1.1055416	1.083330721	2.64E-05	0.002395	Ups
ENSMUSG00000022126	Irg1	9597	4	0.07	1	0.02	0	0	1	0.02	1	0.02	3	0.05	47	0.88	22	0.42	5	0.09	11	0.27	3.5076381	-0.778740502	2.73E-05	0.00246	Ups
ENSMUSG00000044364	Tmem74b	10150	153	2.55	132	2.19	148	2.53	114	2.09	96	1.94	79	1.34	47	0.81	81	1.45	88	1.53	34	0.77	-1.029078	2.328286618	2.85E-05	0.002564	Down
ENSMUSG00000085565	Gm15721	7285	171	2.45	207	2.95	181	2.66	179	2.81	159	2.75	102	1.48	50	0.74	72	1.1	147	2.19	66	1.28	-1.092411	2.789547677	3.11E-05	0.002751	Down
ENSMUSG00000055172	C1ra	10809	61	1.05	72	1.23	78	1.38	82	1.55	80	1.66	189	3.29	332	5.93	271	4.99	149	2.67	67	1.56	1.3395252	2.802740108	3.12E-05	0.002756	Ups
ENSMUSG00000040809	Chil3	20011	9	0.21	3	0.07	8	0.19	1	0.03	0	0	30	0.69	76	1.8	38	0.93	18	0.43	4	0.12	2.8898641	0.03912072	3.32E-05	0.002888	Ups
ENSMUSG00000042265	Trem1	14157	4	0.04	0	0	0	0	0	0	0	0	4	0.04	8	0.08	15	0.16	21	0.21	4	0.05	3.4545802	-1.384774376	3.60E-05	0.003086	Ups
ENSMUSG00000038233	Fam198a	29221	106	1.38	150	1.95	135	1.81	115	1.65	111	1.75	45	0.6	82	1.11	76	1.06	77	1.05	33	0.59	-1.039695	2.274906604	4.26E-05	0.003576	Down
ENSMUSG00000027398	Il1b	6570	11	0.22	9	0.18	17	0.35	17	0.38	9	0.22	50	1.02	73	1.52	57	1.23	23	0.48	15	0.41	1.7028262	0.588010458	4.63E-05	0.003867	Ups
ENSMUSG00000057191	AB124611	19158	15	0.24	7	0.11	7	0.12	8	0.14	7	0.14	28	0.46	86	1.44	33	0.57	36	0.6	8	0.17	2.0240669	0.348160425	4.91E-05	0.004037	Ups
ENSMUSG00000024677	Ms4a6b	14745	46	0.26	33	0.19	27	0.16	28	0.18	21	0.15	52	0.3	244	1.46	85	0.52	126	0.76	36	0.28	1.7429607	1.840820633	5.20E-05	0.004233	Ups
ENSMUSG00000039865	Slc44a3	72877	0	0	1	0.01	1	0.01	0	0	1	0.01	6	0.06	14	0.13	13	0.13	5	0.05	1	0.01	3.3701232	-1.678051533	5.73E-05	0.004635	Ups
ENSMUSG00000029372	Ppbp	1546	1	0.04	0	0	2	0.09	0	0	0	0	11	0.49	14	0.64	1	0.05	8	0.36	5	0.3	3.4095911	-1.673308249	6.17E-05	0.004926	Ups
ENSMUSG00000030560	Ctsc	32804	213	1.49	154	1.07	160	1.15	119	0.92	126	1.07	433	3.07	1474	10.74	488	3.66	277	2.02	108	1.03	1.7679721	4.139895333	6.40E-05	0.005061	Ups
ENSMUSG00000073411	H2-D1	4770	693	10.55	585	8.86	538	8.4	596	9.95	607	11.16	1153	17.77	3258	51.52	1192	19.41	796	12.61	769	15.9	1.1832742	5.683522052	7.46E-05	0.005635	Ups
ENSMUSG00000047139	Cd24a	5094	57	1.49	62	1.61	89	2.38	96	2.75	68	2.14	122	3.22	168	4.55	123	3.43	198	5.38	163	5.77	1.0287875	2.57895821	8.50E-05	0.006265	Ups
ENSMUSG00000032344	Mb21d1	12712	46	0.24	46	0.24	59	0.32	31	0.18	27	0.17	94	0.51	104	0.57	103	0.59	77	0.43	56	0.4	1.0056865	1.735865641	8.71E-05	0.006401	Ups
ENSMUSG00000091996	BC049352	68014	7	0.09	7	0.09	10	0.13	7	0.09	6	0.09	22	0.27	15	0.19	30	0.39	21	0.27	17	0.28	1.4425558	-0.286639304	9.14E-05	0.006654	Ups
ENSMUSG00000060591	Ifitm2	1123	250	18.86	105	7.88	167	12.93	141	11.67	95	8.66	285	21.77	570	44.67	467	37.69	353	27.72	146	14.96	1.202581	3.692146974	9.21E-05	0.006688	Ups
ENSMUSG00000041827	Oasl1	14676	1	0.01	3	0.03	0	0	2	0.02	0	0	2	0.02	92	0.81	7	0.06	11	0.1	5	0.06	4.100246	-0.474188788	9.28E-05	0.006713	Ups
ENSMUSG00000020090	Npffr1	33074	16	0.22	12	0.16	18	0.25	7	0.1	12	0.2	27	0.37	37	0.52	39	0.57	32	0.45	27	0.5	1.2704109	0.324347445	0.000101	0.007151	Ups
ENSMUSG00000004814	Ccl24	3113	1	0.03	1	0.03	3	0.09	1	0.03	0	0	11	0.33	2	0.06	12	0.38	14	0.43	5	0.2	2.6970305	-1.502244425	0.000102	0.007165	Ups
ENSMUSG00000010529	Gm266	1213	86	3.35	210	8.15	67	2.68	65	2.78	42	1.98	36	1.42	56	2.27	25	1.04	23	0.93	17	0.9	-1.619375	1.711632896	0.000103	0.007215	Down
ENSMUSG00000038217	Tlcd2	9185	7	0.11	7	0.11	10	0.16	5	0.09	8	0.15	15	0.24	30	0.5	19	0.32	27	0.45	18	0.39	1.5014462	-0.246493624	0.000107	0.007465	Ups
ENSMUSG00000055632	Hmcn2	146324	31	0.08	29	0.08	50	0.14	37	0.11	21	0.07	106	0.28	68	0.19	68	0.19	79	0.22	45	0.16	1.0624318	1.473127845	0.000108	0.007518	Ups
ENSMUSG00000043789	Vwce	30978	10	0.13	9	0.12	25	0.34	7	0.1	4	0.06	21	0.28	21	0.29	48	0.69	45	0.63	75	1.36	1.9840316	0.564519966	0.000112	0.007713	Ups
ENSMUSG00000024164	C3	24167	7	0.06	10	0.08	5	0.04	2	0.02	1	0.01	17	0.14	59	0.51	26	0.23	16	0.14	8	0.09	2.2572844	-0.222444733	0.000113	0.007795	Ups
ENSMUSG00000047420	Fam180a	13474	63	1.86	20	0.59	63	1.9	50	1.62	35	1.25	34	1.01	13	0.4	15	0.47	15	0.46	7	0.28	-1.52879	0.764676917	0.000116	0.007955	Down
ENSMUSG00000047828	A730018C1	9298	1	0.02	0	0	2	0.04	0	0	2	0.05	17	0.36	0	0	12	0.27	12	0.26	10	0.29	3.1450842	-1.37753046	0.000117	0.008032	Ups
ENSMUSG00000102037	Bcl2a1a	5520	3	0.17	6	0.34	3	0.18	4	0.25	1	0.07	13	0.76	34	2.04	22	1.36	9	0.54	5	0.39	2.1834316	-0.729451094	0.000126	0.008541	Ups
ENSMUSG00000026656	Fcgr2b	17931	166	1.04	86	0.54	84	0.54	75	0.52	66	0.5	213	1.36	456	2.98	212	1.43	239	1.56	94	0.8	1.2839943	3.090140441	0.000131	0.008797	Ups
ENSMUSG00000021998	Lcp1	99742	226	0.81	91	0.33	191	0.7	165	0.65	134	0.58	368	1.34	840	3.14	426	1.64	322	1.2	141	0.69	1.3010566	3.861647209	0.000138	0.009159	Ups
ENSMUSG00000001864	Aif1l	23304	75	0.57	51	0.39	103	0.81	97	0.81	110	1.02	60	0.46	52	0.41	34	0.28	22	0.17	27	0.28	-1.234384	1.742340457	0.000142	0.00932	Down
ENSMUSG00000000204	Slfn4	15036	1	0.01	6	0.04	8	0.06	4	0.03	0	0	14	0.11	452	3.54	22	0.18	3	0.02	2	0.02	4.6093768	1.398317241	0.000144	0.009415	Ups
ENSMUSG00000036587	Fut7	3108	8	0.07	12	0.1	7	0.06	4	0.04	1	0.01	27	0.24	14	0.13	21	0.19	17	0.15	61	0.72	2.1939954	-1.03E-05	0.000147	0.009544	Ups
ENSMUSG00000054362	BC055111	26333	0	0	0	0	1	0.02	1	0.02	0	0	7	0.14	8	0.17	6	0.13	2	0.04	3	0.08	3.2926463	-2.040931133	0.000158	0.010128	Ups
ENSMUSG00000022901	Cd86	62213	57	0.58	50	0.5	55	0.57	61	0.68	46	0.56	71	0.73	147	1.55	123	1.33	93	0.98	136	1.87	1.0734589	2.145039412	0.000159	0.010133	Ups
ENSMUSG00000056498	Tmem154	38384	12	0.11	6	0.05	7	0.07	16	0.16	8	0.09	16	0.15	32	0.3	32	0.31	38	0.36	22	0.27	1.4565037	0.084974855	0.000171	0.010789	Ups
ENSMUSG00000032369	Plscr1	22529	27	0.35	16	0.21	53	0.71	35	0.5	25	0.4	68	0.91	90	1.23	69	0.97	89	1.22	47	0.84	1.1622551	1.441359976	0.000171	0.010789	Ups
ENSMUSG00000026580	Selp	34763	16	0.12	1	0.01	11	0.09	12	0.1	0	0	59	0.46	43	0.35	37	0.31	32	0.26	13	0.14	2.1204792	0.27727501	0.000188	0.011691	Ups
ENSMUSG00000042816	Gpr151	1633	13	0.38	25	0.72	18	0.53	16	0.51	21	0.73	30	0.88	84	2.53	57	1.77	56	1.69	24	0.94	1.3562279	0.87491532	0.00021	0.012792	Ups
ENSMUSG00000016239	Lonrf3	33989	100	1.08	60	0.65	92	1.02	66	0.78	74	0.97	149	1.63	109	1.22	196	2.27	140	1.58	201	2.95	1.0110349	2.634827497	0.000222	0.01343	Ups
ENSMUSG00000026177	Slc11a1	10868	72	0.44	48	0.29	26	0.16	37	0.25	30	0.22	74	0.46	200	1.28	169	1.11	100	0.64	45	0.38	1.3977786	2.034495491	0.000222	0.01343	Ups
ENSMUSG00000040613	Apobec1	24653	43	0.39	39	0.35	34	0.32	28	0.28	25	0.27	51	0.47	283	2.67	84	0.82	64	0.6	55	0.68	1.6205246	1.869934281	0.000246	0.014509	Ups
ENSMUSG00000025473	Adam8	13631	91	0.67	15	0.11	152	1.15	131	1.06	94	0.84	344	2.57	564	4.32	502	3.96	192	1.47	103	1.03	1.7228796	3.449480282	0.000246	0.01454	Ups
ENSMUSG00000024675	Ms4a4c	22477	0	0	1	0.02	0	0	1	0.02	1	0.02	3	0.06	107	2.28	7	0.15	1	0.02	0	0	4.9355914	-0.499355337	0.000256	0.014972	Ups
ENSMUSG00000022221	Ripk3	3862	5	0.11	2	0.04	3	0.07	2	0.05	8	0.2	15	0.32	63	1.38	20	0.45	14	0.31	4	0.11	2.3934136	-0.351794347	0.000286	0.016456	Ups
ENSMUSG00000000386	Mx1	15873	7	0.06	6	0.05	7	0.06	6	0.06	5	0.05	7	0.06	164	1.53	11	0.11	21	0.2	13	0.16	2.7223515	0.426154273	0.000299	0.017048	Ups
ENSMUSG00000092518	Fam71e2	18077	0	0	0	0	0	0	0	0	0	0	2	0.03	2	0.03	4	0.06	5	0.07	2	0.04	4.6181277	-2.493751524	0.000309	0.017584	Ups
ENSMUSG00000046213	Cym	10440	0	0	3	0.11	3	0.12	2	0.08	0	0	13	0.5	2	0.08	6	0.25	13	0.52	21	1.09	2.7294811	-1.232323664	0.000313	0.017716	Ups
ENSMUSG00000091060	7420461P1	11027	8	0.28	0	0	12	0.43	9	0.34	6	0.25	0	0	0	0	1	0.04	0	0	2	0.09	-3.300444	-1.75487749	0.000318	0.017859	Down
ENSMUSG00000059498	Fcgr3	13762	518	6.4	150	1.84	313	3.97	246	3.33	151	2.25	521	6.52	833	10.69	536	7.08	635	8.16	372	6.24	1.0359986	4.429104693	0.000323	0.018078	Ups
ENSMUSG00000029373	Pf4	949	12	0.42	8	0.28	15	0.54	13	0.5	8	0.34	23	0.81	97	3.51	22	0.82	39	1.41	16	0.76	1.7454524	0.457071575	0.000323	0.018078	Ups
ENSMUSG00000030339	Ltbr	7315	186	3.17	157	2.67	124	2.17	106	1.98	98	2.02	286	4.94	495	8.77	243	4.44	255	4.53	130	3.01	1.0084065	3.390915618	0.000349	0.019197	Ups
ENSMUSG00000016496	Cd274	20641	26	0.34	24	0.31	35	0.47	24	0.34	44	0.69	69	0.91	183	2.48	86	1.2	64	0.87	32	0.57	1.4083844	1.6072205	0.000369	0.020029	Ups
ENSMUSG00000031103	Elf4	52087	25	0.11	24	0.1	29	0.13	24	0.11	25	0.13	41	0.18	89	0.4	48	0.22	49	0.22	47	0.28	1.0676597	1.099857025	0.000379	0.020444	Ups
ENSMUSG00000020641	Rsad2	13700	5	0.05	14	0.14	16	0.16	19	0.2	24	0.28	24	0.24	200	2.03	39	0.41	76	0.77	19	0.25	2.0871883	1.193870968	0.000404	0.021576	Ups
ENSMUSG00000047104	Pbp2	564	8	0.67	0	0	5	0.43	11	1.01	7	0.71	1	0.08	0	0	0	0	1	0.09	1	0.11	-3.176171	-1.857193237	0.000406	0.021612	Down
ENSMUSG00000024680	Ms4a2	8197	0	0	0	0	1	0.01	0	0	0	0	2	0.02	3	0.03	5	0.06	2	0.02	10	0.14	3.8501025	-2.183318181	0.000406	0.021612	Ups
ENSMUSG00000085549	1700047F0	69285	45	1.54	58	1.97	32	1.12	21	0.79	42	1.73	25	0.86	23	0.82	14	0.51	18	0.64	7	0.32	-1.254839	0.633610027	0.00041	0.021773	Down
ENSMUSG00000091705	H2-Q2	4521	4	0.08	0	0	2	0.04	0	0	1	0.03	8	0.17	3	0.07	10	0.22	7	0.15	15	0.43	2.5355472	-1.482058212	0.000411	0.021805	Ups
ENSMUSG00000062545	Tlr12	3177	10	0.13	9	0.12	20	0.27	13	0.19	17	0.27	11	0.15	2	0.03	3	0.04	0	0	1	0.02	-2.074639	-0.88768659	0.00042	0.022032	Down
ENSMUSG00000016024	Lbp	26360	103	0.9	10	0.09	77	0.69	49	0.47	31	0.33	114	1.01	258	2.34	149	1.39	169	1.53	102	1.21	1.5180311	2.44274745	0.000452	0.023153	Ups
ENSMUSG00000030413	Pglyrp1	5749	75	5.21	91	6.29	44	3.14	38	2.9	39	3.27	129	9.07	196	14.14	86	6.39	108	7.81	102	9.62	1.0832208	2.231841832	0.000465	0.023687	Ups
ENSMUSG00000050108	Bpifc	52556	2	0.03	3	0.05	4	0.06	0	0	0	0	12	0.19	12	0.19	4	0.07	8	0.13	8	0.17	2.1868	-1.435213624	0.000477	0.02414	Ups
ENSMUSG00000015947	Fcgr1	11061	98	1.59	87	1.4	65	1.08	63	1.12	46	0.9	150	2.46	471	7.93	210	3.64	133	2.24	52	1.14	1.4293417	2.790745399	0.000491	0.02457	Ups
ENSMUSG00000026592	Tex35	9542	8	0.17	6	0.12	4	0.08	2	0.05	8	0.2	22	0.46	9	0.19	6	0.13	13	0.28	141	3.96	2.9249717	0.392515056	0.00052	0.025728	Ups
ENSMUSG00000043740	B430306N0	10884	7	0.03	5	0.02	4	0.02	2	0.01	9	0.05	24	0.12	54	0.28	16	0.08	25	0.13	3	0.02	2.0422453	-0.241559942	0.000532	0.026144	Ups
ENSMUSG00000037336	Mfsd2b	11920	57	0.29	59	0.3	51	0.27	69	0.39	47	0.29	138	0.71	69	0.37	117	0.64	96	0.51	231	1.6	1.2316929	2.320401836	0.000533	0.026144	Ups
ENSMUSG00000051455	Gm1564	18522	44	0.34	32	0.25	23	0.18	34	0.29	21	0.2	77	0.6	39	0.31	64	0.53	65	0.52	121	1.27	1.2716838	1.486335108	0.000558	0.027085	Ups
ENSMUSG00000046694	Fam46b	7807	2	0.04	1	0.02	1	0.02	2	0.05	4	0.1	9	0.19	5	0.11	12	0.27	2	0.04	58	1.63	3.1587031	-0.698339592	0.00056	0.02713	Ups
ENSMUSG00000101389	Ms4a4a	17268	4	0.23	2	0.11	2	0.12	5	0.31	2	0.14	9	0.52	31	1.82	8	0.48	7	0.41	10	0.77	2.033954	-0.977440514	0.000584	0.028041	Ups
ENSMUSG00000012123	Aim1l	26593	25	0.13	29	0.14	30	0.15	35	0.19	23	0.14	59	0.3	33	0.17	75	0.4	59	0.31	105	0.72	1.2314311	1.353738279	0.000585	0.028082	Ups
ENSMUSG00000047884	Klk9	4866	4	0.13	2	0.06	2	0.07	0	0	1	0.04	17	0.55	51	1.7	34	1.17	0	0	1	0.04	3.3499686	-0.602319887	0.000667	0.030963	Ups
ENSMUSG00000002910	Arrdc2	4566	301	6.11	136	2.75	259	5.4	266	5.93	193	4.73	382	7.86	197	4.16	524	11.39	619	13.09	846	23.33	1.1823857	4.292707946	0.000692	0.031903	Ups
ENSMUSG00000070369	Itgad	69441	12	0.11	5	0.05	5	0.05	6	0.06	6	0.07	8	0.07	12	0.11	27	0.26	24	0.23	50	0.62	1.8703889	-0.133900676	0.000711	0.032613	Ups
ENSMUSG00000052861	Dnah6	204046	17	0.06	36	0.12	28	0.1	23	0.09	34	0.14	68	0.24	79	0.28	75	0.28	59	0.21	26	0.12	1.0633922	1.222802259	0.000771	0.034717	Ups
ENSMUSG00000032092	Mpzl2	11591	71	1.08	18	0.27	92	1.44	53	0.89	25	0.46	107	1.65	151	2.39	112	1.82	135	2.14	98	2.03	1.195911	2.154880142	0.000773	0.034722	Ups
ENSMUSG00000074449	Gm15319	25547	12	0.23	16	0.3	4	0.08	20	0.41	20	0.45	61	1.16	21	0.41	42	0.84	66	1.29	23	0.59	1.4559879	0.61592798	0.000808	0.035888	Ups
ENSMUSG00000063193	Cd300lb	11606	1	0.01	1	0.01	0	0	0	0	2	0.03	2	0.03	40	0.56	9	0.13	3	0.04	1	0.02	3.4939688	-1.326406934	0.000868	0.037971	Ups
ENSMUSG00000023224	Serping1	10058	144	2.41	61	1.01	170	2.91	120	2.2	101	2.04	202	3.42	534	9.26	296	5.29	234	4.07	112	2.54	1.1405743	3.317121299	0.000877	0.038314	Ups
ENSMUSG00000055102	Zfp819	11117	0	0	2	0.02	1	0.01	0	0	1	0.01	5	0.04	2	0.02	8	0.07	2	0.02	19	0.2	3.0652058	-1.69222115	0.000905	0.03937	Ups
ENSMUSG00000025461	Cd163l1	12879	0	0	3	0.04	3	0.04	0	0	0	0	6	0.08	18	0.26	9	0.13	4	0.06	3	0.06	2.5705719	-1.584921296	0.000934	0.040268	Ups
ENSMUSG00000026390	Marco	30487	0	0	0	0	0	0	0	0	0	0	2	0.05	8	0.2	1	0.03	0	0	8	0.26	5.0463503	-2.325466464	0.000941	0.040465	Ups
ENSMUSG00000024905	Mtl5	18967	4	0.03	1	0.01	0	0	6	0.05	0	0	7	0.06	13	0.11	9	0.08	7	0.06	14	0.16	2.1236227	-1.272961638	0.000962	0.041215	Ups
ENSMUSG00000031212	Pgr15l	9509	2	0.02	0	0	3	0.03	1	0.01	2	0.02	9	0.08	3	0.03	4	0.04	6	0.05	30	0.36	2.7113159	-1.265516007	0.000986	0.042032	Ups
ENSMUSG00000017697	Ada	23656	14	0.31	15	0.33	6	0.14	22	0.53	12	0.32	24	0.54	30	0.69	52	1.23	28	0.65	34	1.02	1.2396069	0.390926857	0.001024	0.043155	Ups
ENSMUSG00000052749	Trim30b	14503	3	0.01	8	0.04	6	0.03	3	0.02	0	0	16	0.08	14	0.07	20	0.1	14	0.07	5	0.03	1.6977622	-0.869915683	0.001034	0.043449	Ups
ENSMUSG00000044086	Lmod3	14253	0	0	0	0	0	0	0	0	0	0	1	0.02	3	0.07	3	0.07	2	0.04	4	0.11	4.4514554	-2.572885982	0.001054	0.044171	Ups
ENSMUSG00000028885	Smpdl3b	24287	29	0.68	69	1.62	45	1.09	17	0.44	17	0.48	78	1.86	160	3.92	71	1.79	55	1.35	78	2.5	1.3087709	1.695589902	0.00113	0.046348	Ups
ENSMUSG00000030219	Erp27	14870	1	0.01	1	0.01	0	0	2	0.03	2	0.03	5	0.07	1	0.01	8	0.12	9	0.13	14	0.26	2.512777	-1.630454022	0.001173	0.047509	Ups
ENSMUSG00000097182	A830009L0	19096	281	4.74	431	7.23	210	3.63	224	4.14	188	3.83	209	3.57	207	3.62	112	2.02	115	2.02	45	1.03	-1.036748	3.364133165	0.001178	0.047576	Down
ENSMUSG00000038521	C1s1	12015	49	0.45	52	0.48	38	0.36	40	0.41	41	0.46	122	1.14	113	1.09	151	1.49	72	0.69	30	0.38	1.0546802	1.856117236	0.00119	0.047874	Ups
ENSMUSG00000041423	Paqr6	3958	62	0.9	104	1.5	117	1.73	80	1.27	69	1.2	35	0.51	22	0.33	39	0.6	67	1.01	49	0.96	-1.042031	1.768055095	0.001205	0.048318	Down
ENSMUSG00000026831	1700007K1	4332	18	0.64	31	1.1	21	0.77	29	1.13	29	1.24	97	3.49	55	2.03	71	2.7	38	1.41	28	1.35	1.0791885	1.130829785	0.001242	0.049608	Ups
