# Supplementary figures and images for: Microglial TREM-1 receptor mediates neuroinflammatory injury via interaction with SYK in experimental ischemic stroke
Source: Cell Death Dis. 2019 Jul 19;10(8):555. doi: 10.1038/s41419-019-1777-9 (PMC6642102; doi:10.1038/s41419-019-1777-9)

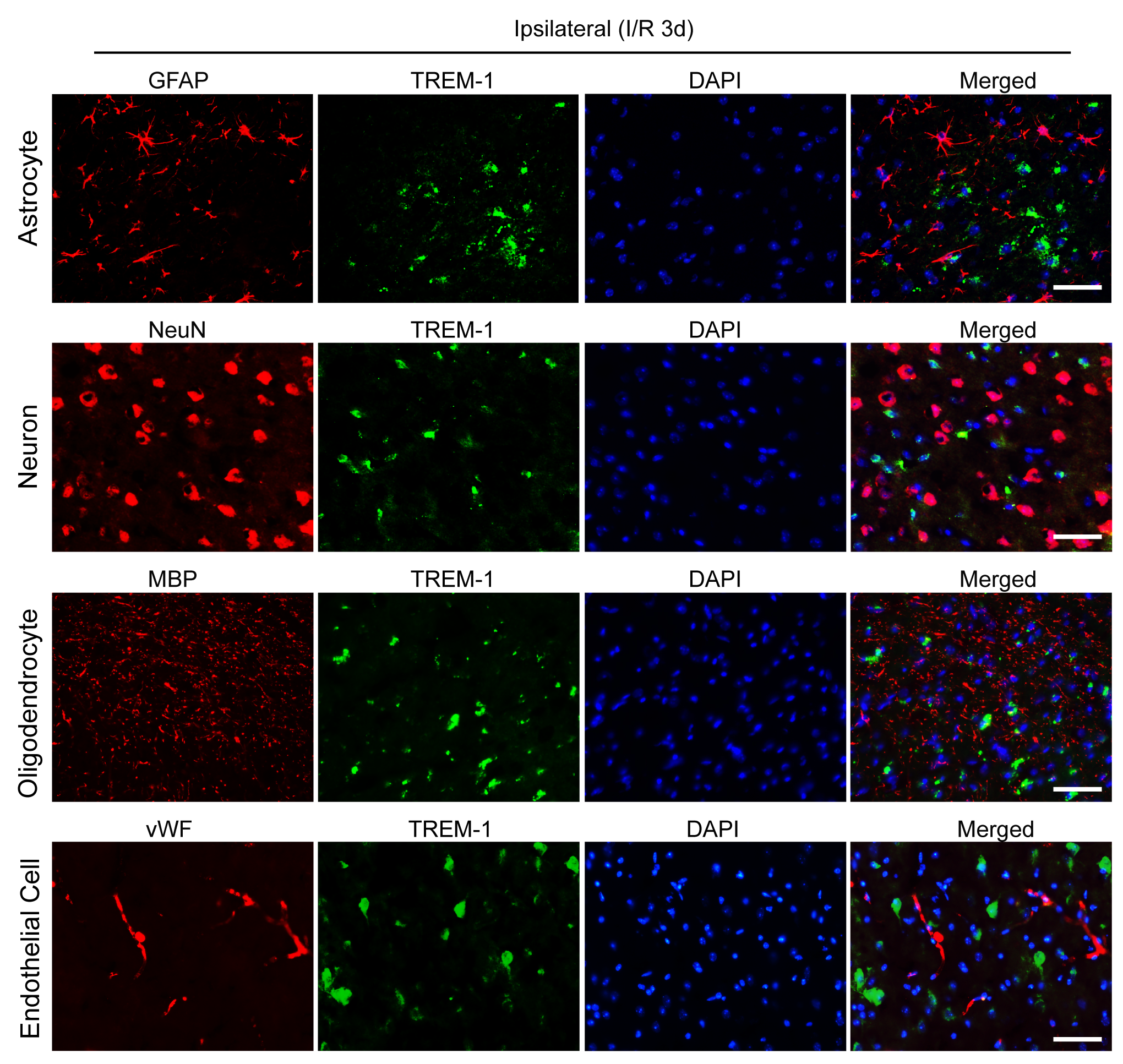

Supplement: Supplementary file 5 — Supplementary Figure S2 [file 41419_2019_1777_MOESM5_ESM.tif]

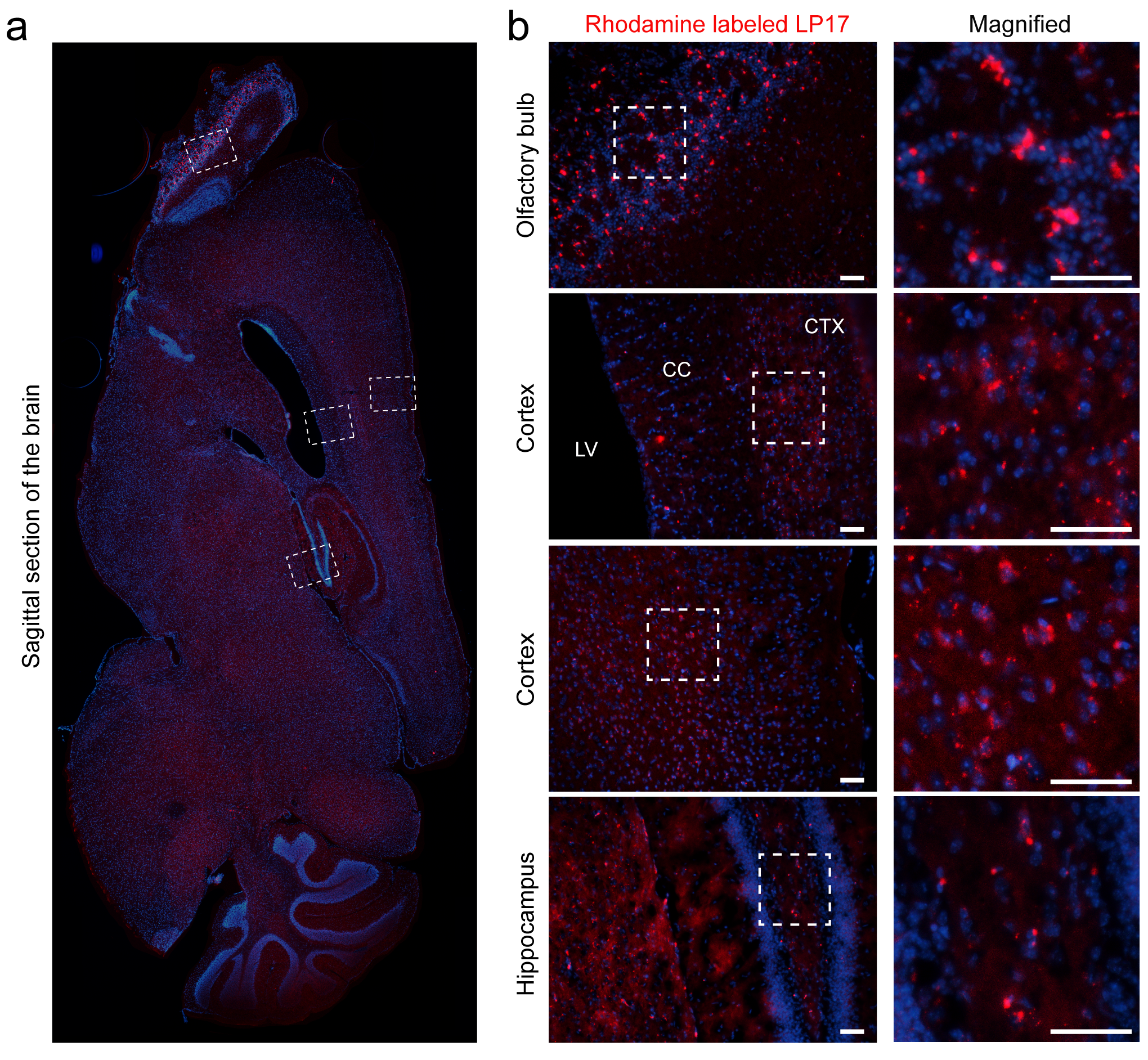

Supplement: Supplementary file 6 — Supplementary Figure S3 [file 41419_2019_1777_MOESM6_ESM.tif]

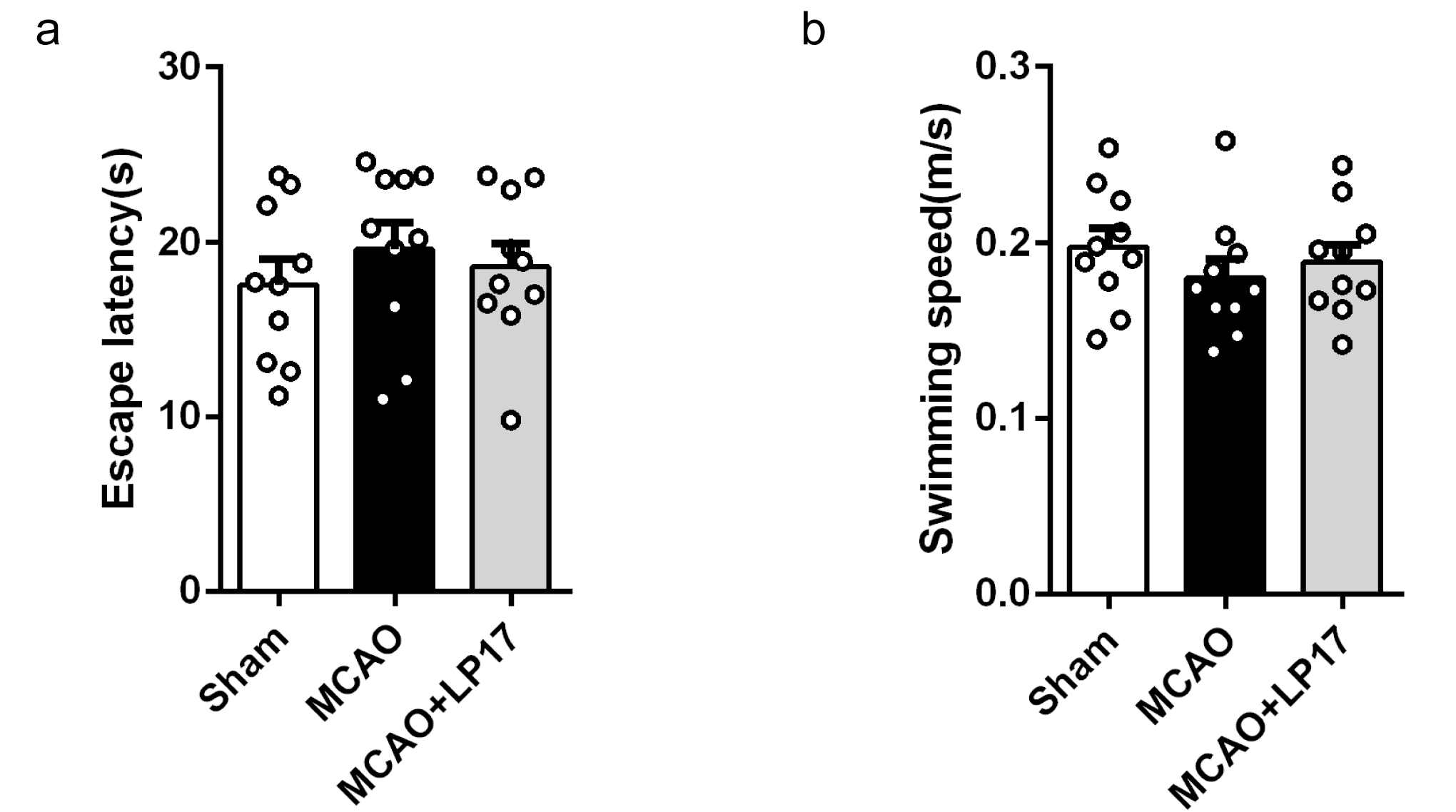

Supplement: Supplementary file 7 — Supplementary Figure S4 [file 41419_2019_1777_MOESM7_ESM.tif]

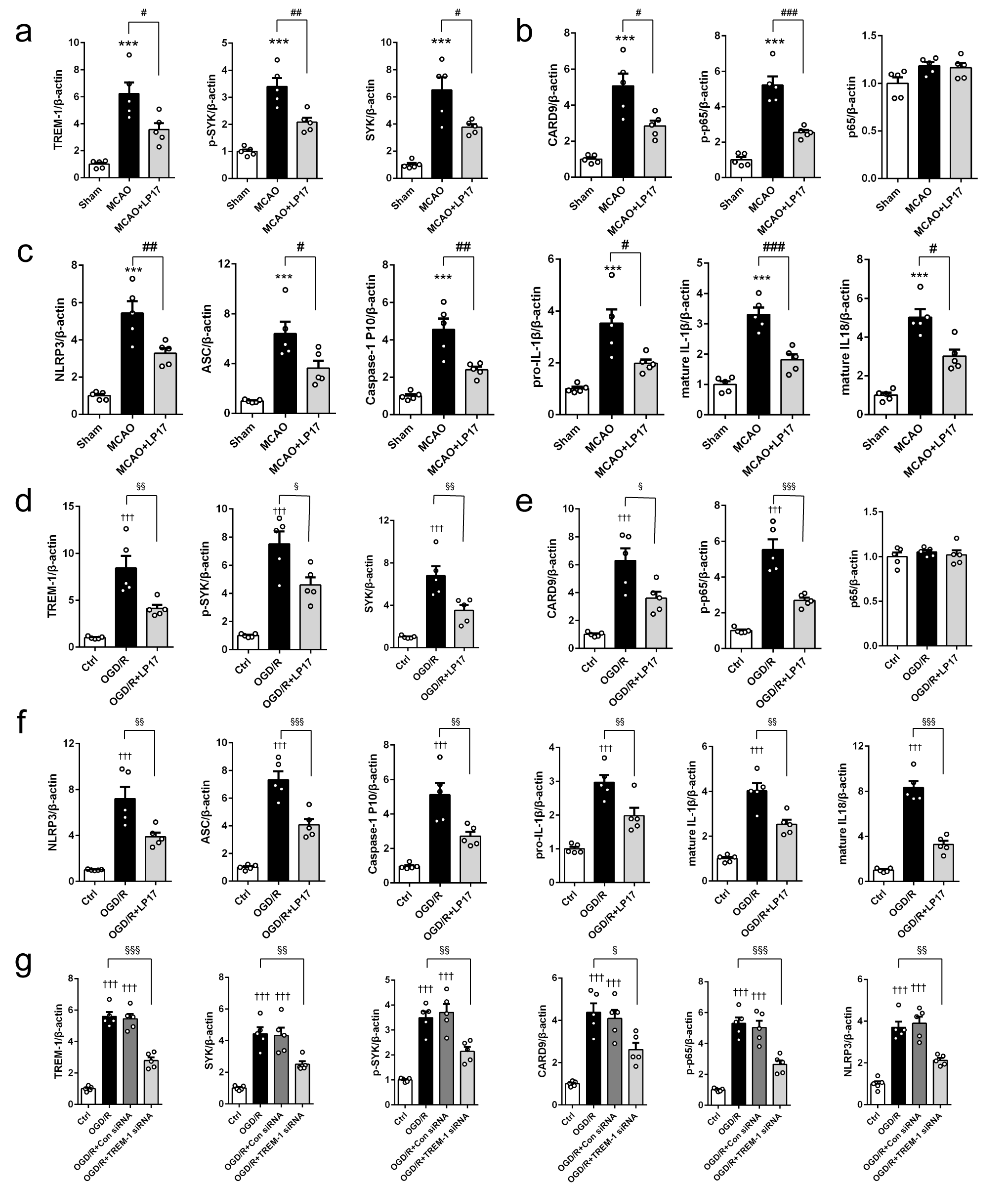

Supplement: Supplementary file 8 — Supplementary Figure S5 [file 41419_2019_1777_MOESM8_ESM.tif]

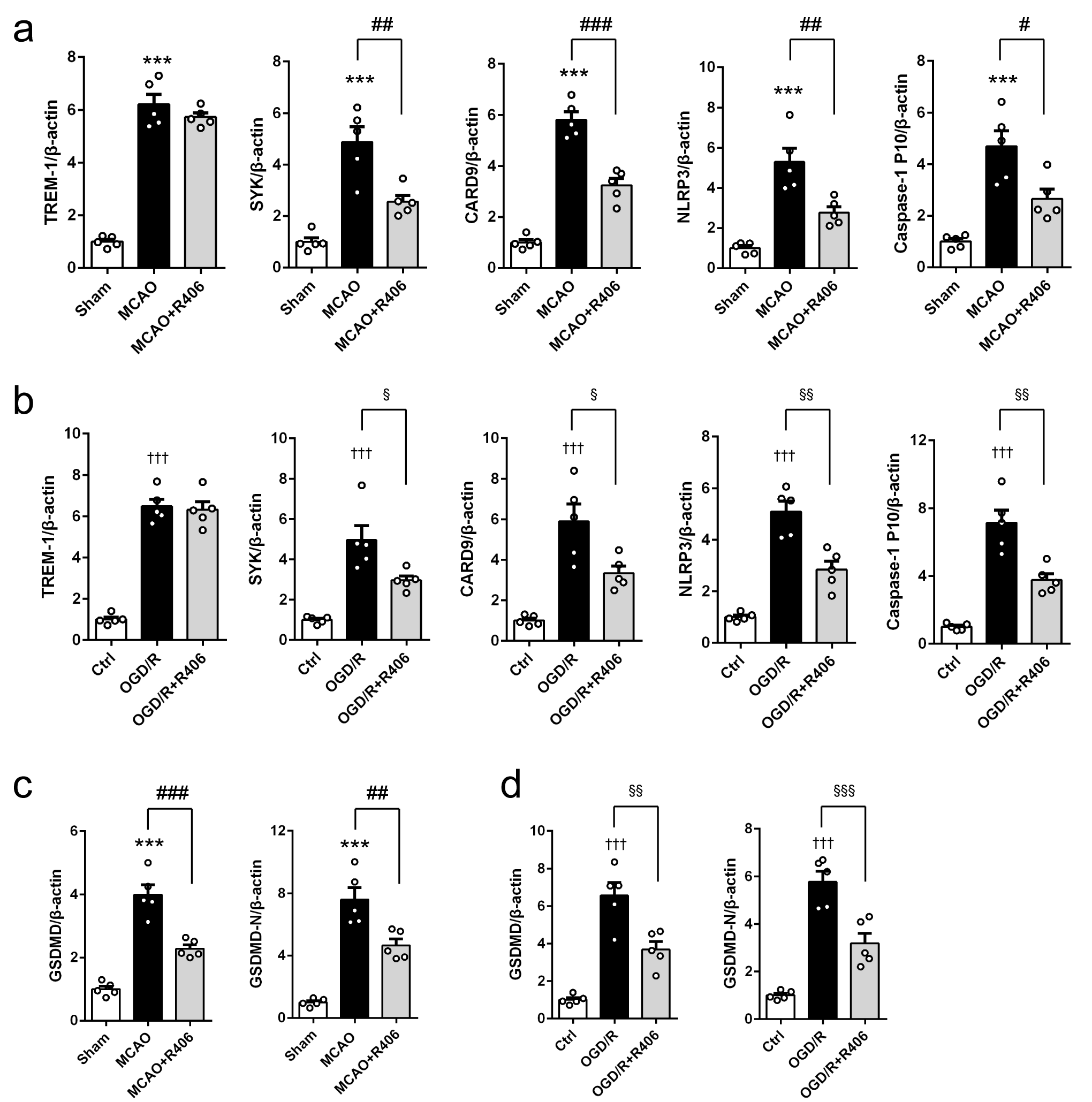

Supplement: Supplementary file 9 — Supplementary Figure S6 [file 41419_2019_1777_MOESM9_ESM.tif]

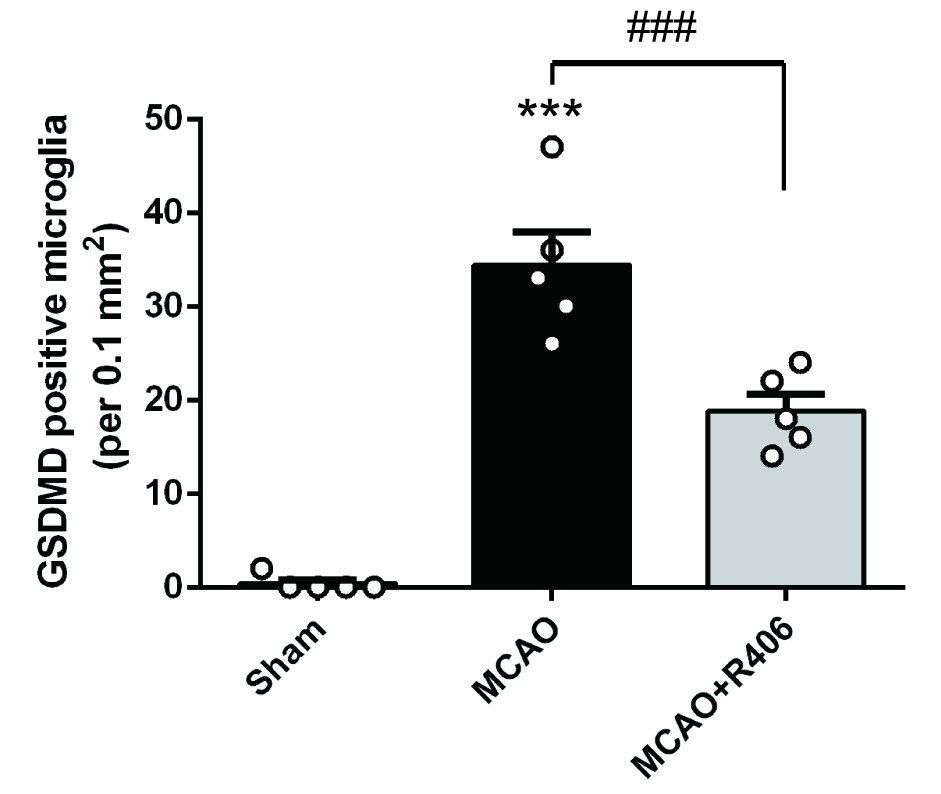

Supplement: Supplementary file 10 — Supplementary Figure S7 [file 41419_2019_1777_MOESM10_ESM.tif]
